# Biological Activity of Plant-Derived Peptides, Hydrolysates and Digests in the Caco-2 Intestinal Epithelial Model: A Systematic Critical Review

**DOI:** 10.3390/ijms27177905

**Published:** 2026-09-04

**Authors:** Simone Perna, Mattia Di Nunzio

**Affiliations:** Department of Food, Environmental and Nutritional Sciences (DeFENS), Università degli Studi di Milano, 20133 Milan, Italy

**Keywords:** plant proteins, plant food, gut, antioxidant, anti-inflammatory, antihypertensive, antidiabetic, Caco-2 cells, protein hydrolysate, structure–activity relationship

## Abstract

Plant-derived bioactive peptides are widely investigated for their effects on intestinal epithelial function using Caco-2 cells, with reported antioxidant, anti-inflammatory, antihypertensive and antidiabetic activities. However, this literature remains heterogeneous and has rarely addressed whether the tested material corresponds to the actual bioactive species. This PRISMA-compliant systematic review searched PubMed and Scopus for studies on plant-derived peptides, hydrolysates or digests in Caco-2 cells, identifying 34 eligible studies. The material tested in each study was classified a priori as a purified or synthetic single peptide, an ultrafiltration or chromatographic fraction, a whole hydrolysate or gastrointestinal digest, or an intact parent protein used as a comparator, because these forms do not support equivalent structure–activity inference. Risk of bias was assessed using the QUIN tool, adopted in the absence of an instrument validated for nutritional cell-culture research. For each study, metabolic characterisation of the tested peptide and the physiological relevance of the concentrations used were evaluated. Directionally consistent bioactivity, including antioxidant, anti-inflammatory, antihypertensive and antidiabetic effects, was observed across most functional domains, although effect magnitude and stress dependence varied considerably. Mass-based concentrations did not uniformly exceed a conservative exploratory luminal benchmark (0.1–1 mg/mL), but upper-range experiments exceeded it by approximately 25-fold in the antioxidant domain, 50-fold in the anti-inflammatory and antidiabetic domains, and 500-fold in the viability domain; in vivo confirmation was available for a single identified metabolite (LTFPG). Because the primary studies did not report effect estimates with measures of precision, the synthesis is qualitative, based on vote counting by direction of effect, and no pooled effect sizes, heterogeneity statistics or certainty ratings were derived. Overall, plant-derived peptides show directionally consistent bioactivity in Caco-2 models, but the evidence rarely identifies the responsible molecular species or confirms physiological attainability. Future work should prioritise metabolic characterisation, exposure-justified concentrations, and multi-omics approaches to clarify underlying mechanisms.

## 1. Introduction

Over the past decades, growing epidemiological and experimental evidence has highlighted the association between plant-based dietary patterns and a reduced incidence of chronic non-communicable diseases, including type 2 diabetes mellitus, cardiovascular disease, obesity, and colorectal cancer [1]. In this framework, plant-derived bioactive peptides have emerged as key functional components potentially mediating part of the health-promoting effects attributed to plant-rich diets, exerting a broad spectrum of biological activities, encompassing antioxidant, anti-inflammatory, antihypertensive, and antidiabetic effects [2].

Plant proteins represent an abundant and structurally diverse source of bioactive peptides, encrypted within the primary sequence of storage and structural proteins found in legumes, cereals, oilseeds, and other plant-based food matrices. These peptides, typically ranging from 2 to 20 amino acids in length, remain inactive within the intact protein structure and are released upon enzymatic hydrolysis during gastrointestinal digestion or food processing, including fermentation and enzymatic treatment [3,4]. The biological activity of the released sequences is strictly dependent on their amino acid composition, chain length, and three-dimensional conformation, which collectively determine their interaction with specific molecular targets [5].

Upon oral ingestion, dietary peptides encounter the gastrointestinal tract as the first and most complex biological interface between the external environment and the host organism. Intestinal cells, and particularly the enterocytes lining the epithelial monolayer, represent the primary biological barrier through which dietary peptides interact with the host, making them a critical site for the initial modulation of biological responses. At this level, bioactive peptides may act through multiple mechanisms, including direct interaction with membrane-bound receptors and enzymes, modulation of intracellular signalling pathways, and, more recently, nutrigenomic mechanisms involving the modulation of gene and protein expression profiles [6].

Although human intervention trials constitute the gold standard for establishing causal relationships between dietary components and health outcomes, the development of well-validated in vitro models enables the systematic investigation and mechanistic characterisation of the underlying cellular and molecular pathways. Such models represent an essential and indispensable preliminary step in the scientific evaluation of the health-promoting properties of food and food-derived bioactive compounds [7].

Among the available experimental models for studying peptide–intestinal cell interactions, the Caco-2 cell line has established itself as the most widely adopted and well-characterised in vitro system for mimicking the human intestinal epithelium. Caco-2 cells are derived from a human colorectal adenocarcinoma but, upon reaching confluency, undergo spontaneous differentiation into a polarised monolayer that closely recapitulates the morphological and functional features of mature small intestinal enterocytes [8]. Despite certain limitations, including the absence of mucus-secreting goblet cells and immune components, Caco-2 monolayers remain the gold-standard in vitro tool for mechanistic studies on the biological activity of dietary bioactive compounds at the intestinal level.

Despite two decades of accumulating reports, three specific gaps remain unaddressed by the existing narrative and systematic reviews of this field, and together they define the contribution of the present work. First, previous syntheses have catalogued reported activities without asking whether the molecular species responsible for a measured effect is in fact the material added to the medium. Differentiated Caco-2 monolayers express a complete brush-border peptidase complement, so a supplemented sequence may be cleaved before or during target engagement, and a structure–activity conclusion drawn from the nominal sequence may therefore be attributed to the wrong molecule. Second, the concentrations at which effects are demonstrated have almost never been compared with any estimate of the exposure achievable at the intestinal epithelium after a realistic meal, so that in vitro potency and dietary plausibility have remained unconnected. Third, activities obtained with purified sequences, with ultrafiltration or chromatographic fractions, with whole hydrolysates and gastrointestinal digests, and with intact parent proteins have been pooled under the single label of bioactive peptides, although these materials do not support equivalent mechanistic inference. Accordingly, this review does not merely update the catalogue of reported effects: it appraises, study by study, whether the bioactive species was identified, whether the tested concentration is plausibly attainable in the gut lumen, and which class of material was actually assayed, and it uses these three axes to convert a heterogeneous body of positive findings into an explicit research agenda.

The present review systematically examines the biological and health-promoting effects of plant-derived bioactive peptides, hydrolysates and gastrointestinal digests in the Caco-2 cell line. The scope is deliberately restricted to Caco-2 monolayers, which are by a wide margin the most widely adopted intestinal cell model and the only one for which a body of evidence sufficient to support a systematic appraisal currently exists; studies performed exclusively in other intestinal cell systems, in co-cultures or in organoids were not eligible, and the consequences of this restriction are addressed in Section 3.12. Evidence from in vitro studies conducted on Caco-2 demonstrated that plant-derived peptides can modulate key cellular processes and conditions, such as cell viability, inflammation, oxidative stress, and the activity of enzymes involved in metabolic regulation, including dipeptidyl peptidase-IV (DPP-IV) and angiotensin-converting enzyme (ACE).

Furthermore, this review examines the intrinsic limitations and critical issues stemming from the existing literature, which complicates the drawing of coherent and reliable conclusions. Accordingly, potential strategies are proposed to address these shortcomings and to consolidate the most appropriate experimental parameters into a unified, comprehensive framework.

## 2. Materials and Methods

### 2.1. Data Extraction

This systematic review was designed, conducted and reported according to the Preferred Reporting Items for Systematic Reviews and Meta-Analyses (PRISMA) guidelines [9]. Reporting followed the updated PRISMA 2020 statement [10], and the completed PRISMA 2020 checklist is provided as Table S1. The search was carried out by using the PubMed and Scopus databases on 8 October 2025 and was conducted using the following keywords and Boolean operators: “food bioactive peptides” AND “cultured cells”. The quotation marks were entered as phrase-search operators, and the exact strings submitted to each interface, together with the fields interrogated, the filters and date limits applied, the date of execution and the number of records retrieved, are reproduced verbatim as Table S5 so that both searches can be replicated. In PubMed, the string was entered in the basic search box as “food bioactive peptides” AND “cultured cells”, with no field tags, no filters and no publication-date restriction. PubMed resolves a quoted string as an exact phrase only when that string is present in its phrase index; a quoted string that is absent from the index is dissolved into its component terms, which are then processed by Automatic Term Mapping and combined with the Boolean AND operator. The two quoted concepts were therefore not handled identically by the interface, and the query behaved in part as a phrase search and in part as a term intersection. This is the reason a deliberately narrow-looking string returned 1552 records rather than the small set that strict phrase matching on both concepts would have produced, and the translation returned by PubMed in the Search Details field of the search history is reproduced in full in Table S5. In Scopus, the same concepts were entered as TITLE-ABS-KEY (“food bioactive peptides” AND “cultured cells”), restricting retrieval to the title, abstract and keyword fields, with no further limits; this returned 45 records. In Scopus, double quotation marks denote a loose phrase search, in which the enclosed terms are matched adjacently but punctuation and lexical variants are disregarded, whereas braces denote exact-string matching; braces were not used, so retrieval in Scopus rested on loose rather than strict phrase matching. The second search, run on 4 May 2026, used the identical strings in both databases with the publication-date range set from 9 October 2025 onwards and no other change. Neither controlled vocabulary (MeSH in PubMed, index keywords in Scopus) nor truncation, proximity or synonym expansion was applied, a decision whose consequences for sensitivity are considered in Section 3.13. Inclusion criteria were: (i) use of the English language; (ii) biological effects, measured in Caco-2 cells, of material derived from plant foods, ingredients, by-products or proteins, in any of four forms, namely purified or synthetic single peptides, ultrafiltration or chromatographic fractions, whole protein hydrolysates or simulated gastrointestinal digests, and intact parent proteins when these were tested as comparators for one of the preceding forms. Intact parent proteins were eligible only in this comparator role, because a study contrasting a protein with its digest bears directly on the identity of the bioactive species, which is a primary question of this review; such studies are labelled accordingly throughout, and their results are never used to support a peptide-attributed conclusion. Exclusion criteria were: (i) plant-based food incorporated in potentially animal-based food; (ii) reviews; (iii) studies whose sole objective was the quantification of transepithelial peptide transport, permeability or apparent permeability coefficients, with no biological-activity endpoint, whereas studies reporting a biological-activity endpoint alongside transport measurements were retained and only their bioactivity data were extracted; (iv) studies in which the reported outcomes were generated exclusively in a co-culture system and could therefore not be attributed to Caco-2 cells alone, whereas for studies reporting a Caco-2 monoculture arm alongside a co-culture arm only the monoculture data were extracted; (v) inadequate statistical reporting, operationally defined as the occurrence of any of the following: no inferential test reported for the comparison between control and treated conditions; no measure of dispersion reported for the compared groups; the number of independent biological replicates not stated, or fewer than three; or a claim of statistical significance without identification of the test applied; (vi) lack of detailed explanations that allowed the experimental conditions to be fully understood; (vii) either genetically modified Caco-2 cells or modified peptides. The review protocol was not prospectively registered, and a formal written protocol was not prepared; the eligibility criteria and the extraction and appraisal procedures were agreed between the two reviewers before screening began and are reported in full as Protocol S1. The absence of prospective registration is a substantive limitation of this review and is discussed as such in Section 3.13.

**Figure 1:**
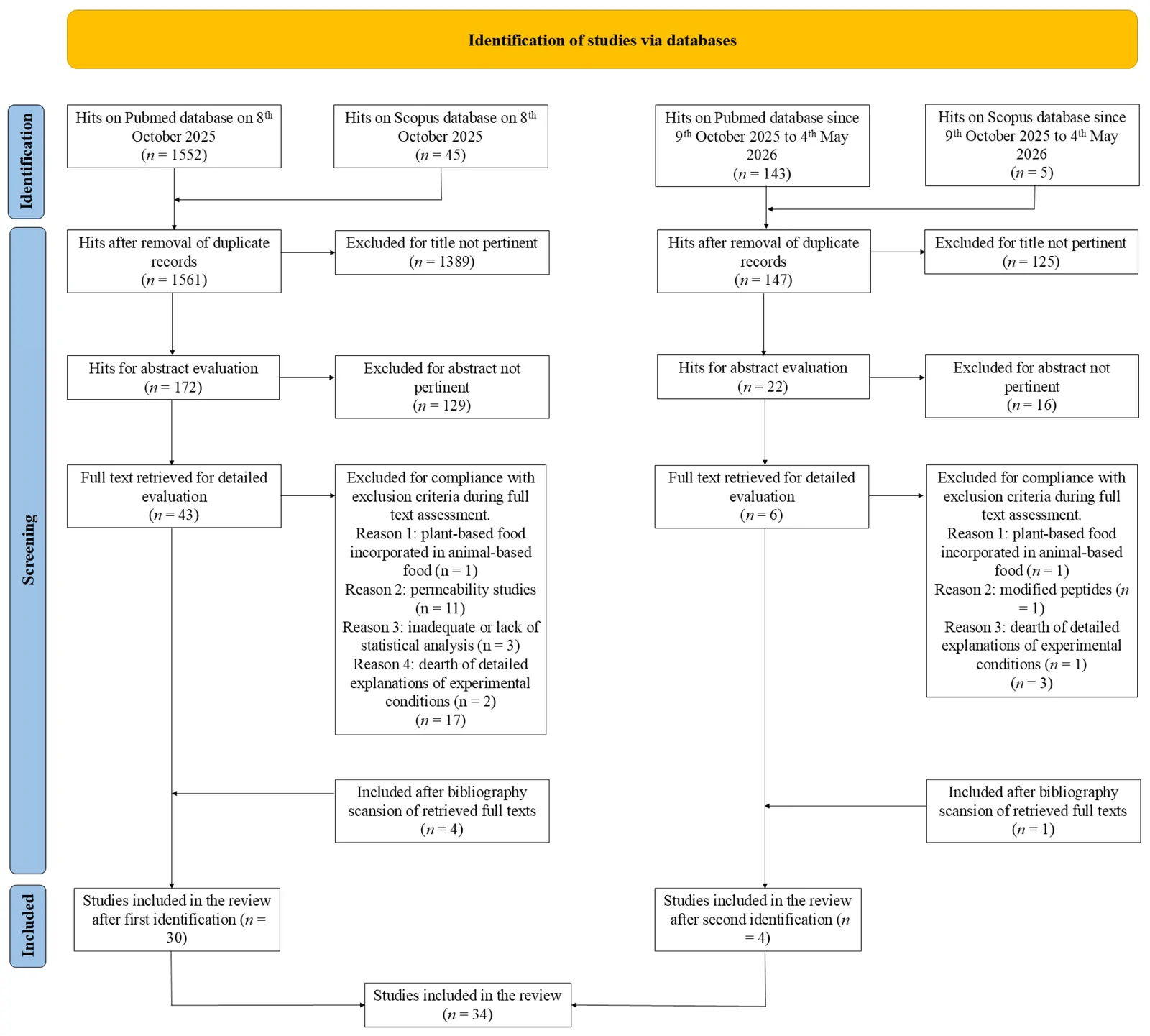
Flow chart of papers included in the review.

Articles retrieved through the systematic search were assigned to two reviewers (S.P. and M.D.N.), who first assessed the consistency of the title and abstract with the review’s objective and inclusion criteria, and then examined the full text of all potentially relevant publications. Any disagreements were resolved through constructive discussion without using any software. The first search returned 1552 records from PubMed and 45 from Scopus, and 1561 records remained after removal of duplicates. During the screening process (reviewing titles), 1389 records were excluded, leaving 172 records for abstract evaluation. After abstract analysis, another 129 articles were excluded. Of the 43 full texts retrieved for detailed evaluation, 17 studies were excluded for non-compliance with the inclusion criteria or compliance with the exclusion criteria during detailed evaluation of the full text. Each of these 17 records, together with the 3 records excluded at the full-text stage of the second search described below, is listed individually with its reason for exclusion in Table S3. Four articles were included after bibliography scansion of the retrieved full texts. These studies were identified by manually scanning the reference lists of all articles included after full-text screening, with each retrieved record subsequently assessed against the same predefined inclusion and exclusion criteria applied to the primary database search, and this backward citation searching was performed during the full-text screening phase without forward citation searching, and all records identified through this supplementary procedure were screened in duplicate and reported separately in the PRISMA flow diagram to ensure transparency and reproducibility of the search process. Altogether, 30 records, comprising the 26 retained after full-text assessment plus the 4 identified through citation chasing, were selected in this first search. A second search was carried out on 4 May 2026 using the same keywords and Boolean operators with the selected publication period starting on 9 October 2025. The second search returned 143 records from PubMed, 5 from Scopus, and 147 records remained after removal of duplicates. Analysis of titles and abstracts excluded 141 records. The same selection process as in the first search was applied, and of the 6 records retrieved for detailed evaluation, 3 were excluded for non-compliance with the inclusion criteria or compliance with the exclusion criteria. One article was included after bibliography scansion of the retrieved full text. The 4 records selected after the second search resulted in a total of 34 records being included in this review. Details of the selection process are presented in Figure 1.

No automation tools, machine-learning classifiers or text-mining software were used at any stage of screening or data extraction. Data were extracted independently and in duplicate by the same two reviewers using a purpose-built extraction form, reproduced as Table S2, piloted on five randomly selected studies; discrepancies were resolved by discussion, and the original investigators were not contacted for additional information.

The outcomes sought were antioxidant activity, inflammatory response, antihypertensive activity, antidiabetic activity, other biological effects, and cell viability or apoptosis. All results compatible with each outcome domain were extracted, and a study reporting outcomes in more than one domain contributes to each of them. For every study we additionally extracted the plant source and food matrix, the form of the tested material (isolated peptide, protein hydrolysate, or in vitro digest), the peptide sequence where identified, the digestion protocol, the tested concentration and the unit in which it was expressed, the exposure duration, the applied stressor where present, the assay used, and the direction of the reported effect. No assumptions were made regarding missing or unclear information: studies providing insufficient methodological detail to reconstruct the experimental conditions were excluded a priori (exclusion criterion vi).

### 2.2. Risk of Bias Assessment

The risk of bias was assessed by means of the QUIN tool (risk-of-bias tool for assessing in vitro studies conducted in dentistry) [11]. The quality of each study was assessed according to a predetermined set of domains (clearly stated aims/objectives; detailed explanation of sample size calculation; detailed explanation of sampling technique; details of comparison group; detailed explanation of methodology; operator details; randomisation; method of measurement of outcome; outcome assessor details; blinding; statistical analysis; presentation of results). Each criterion is assigned a score ranging from 0 to 2, with 0 representing ‘not specified’, 1 representing ‘inadequately specified’, 2 representing ‘adequately specified’, and N.a. representing ‘not applicable’. The final score is determined by the following formula: final score = (total score × 100)/(2 × number of criteria applicable). The final score is used to determine the article’s risk of bias, with the following categories: low risk (>70%), medium risk (50–70%), and high risk (<50%). The assessment was conducted independently by both reviewers, and any uncertainties or disagreements were resolved through discussion. As noted in Section 3.1, the majority of QUIN items interrogate the completeness of methodological reporting rather than the presence of a demonstrated bias, so that the instrument quantifies reporting quality and, only indirectly, risk of bias. The resulting scores are accordingly interpreted throughout this review as risk of bias arising from incomplete reporting, and not as direct measures of internal validity. The per-study scoring worksheet is provided as Table S4 and the corresponding traffic-light plot as Figure S1. The convention adopted for the domains whose applicability to cell-culture work is limited requires an explicit statement. Only one domain, sampling technique, was scored N.a. and thereby removed from the denominator, because a Caco-2 experiment does not draw a sample from a population and the domain has no operational meaning in this setting. The four domains of randomisation, blinding, operator details and outcome-assessor details were instead scored 0, that is, not specified, and were retained in the denominator. This was a deliberate choice, and it rests on the view that these four domains are not strictly inapplicable to in vitro work: the allocation of wells or plates to treatment can be randomised, the analyst reading a plate, quantifying an image or scoring a blot can be blinded to treatment identity, and the identity and experience of the operator can be reported. All three practices are recommended for preclinical laboratory research and reduce observer-driven bias in measurements that involve subjective or semi-quantitative judgement. Their complete absence from all 34 included studies is therefore an informative property of this literature rather than an artefact of instrument mismatch, and scoring them N.a. would have removed that information from the appraisal. Because the QUIN denominator counts only applicable criteria, this decision is consequential and was examined in a sensitivity analysis. With the four domains scored 0, eleven criteria are applicable and the denominator is 22, which yields the reported range of 50.0% to 63.6% and a uniform medium-risk classification. Reclassifying all four as N.a. would leave seven applicable criteria and a denominator of 14; since domains scored 0 contribute nothing to the numerator, the total scores would be unchanged and the recomputed percentages would rise to between 78.6% and 100%, moving every included study into the low-risk category. The overall classification is thus wholly dependent on this convention. We retain the conservative scoring, because a body of literature in which no study reports randomisation, blinding or operator identity should not be described as being at low risk of bias by virtue of a denominator adjustment; the alternative computation is reported here so that readers can judge the effect of the other convention for themselves.

### 2.3. Effect Measures and Synthesis Methods

Studies were assigned to one or more functional domains—antioxidant, anti-inflammatory, antihypertensive, antidiabetic, other biological effects, and cell viability or apoptosis—on the basis of the outcome measured, so that a study reporting several outcomes contributes to more than one domain. No pooled effect measures were computed. Results are presented as the direction of effect relative to each study’s own control condition (increase, decrease, or no significant change), together with the quantitative values reported by the original authors, such as IC_50_ values or percentage changes, reproduced as given. No data conversion or imputation was performed; concentrations are reported in the units used by the original authors, and mass-based and molar concentrations were not inter-converted because the molecular identity of the tested material was undefined in most hydrolysate and digest studies.

Two classifications were applied to every included study and are used throughout the synthesis. The first concerns the form of the tested material and distinguishes purified or synthetic single peptides (PPs), ultrafiltration or chromatographic fractions (FRs), whole protein hydrolysates and simulated gastrointestinal digests (HDs), and intact parent proteins (IPs); a study testing more than one form was assigned all applicable labels. The second concerns the characterisation status of the bioactive species and assigns each study to exactly one of seven mutually exclusive categories: (A) a breakdown metabolite was identified and independently tested; (B) an uncharacterised fraction or hydrolysate was tested and the active component was not isolated; (C) a single purified or synthetic peptide was tested directly, with no assessment of its stability in the assay system; (D) a simulated gastrointestinal digestion was applied but brush-border-generated products were not characterised; (E) the tested sequence is characterised in the independent literature as resistant to intestinal digestion; (F) an intact parent protein was the primary material tested; (G) an intact protein and its gastrointestinal digest were directly compared. Where a study satisfied more than one description, it was assigned to the category reflecting the most advanced level of characterisation achieved. Both classifications were applied independently by the two reviewers; disagreements were resolved by discussion, and the resulting assignments are reported in Table 1.

**Figure 2:**
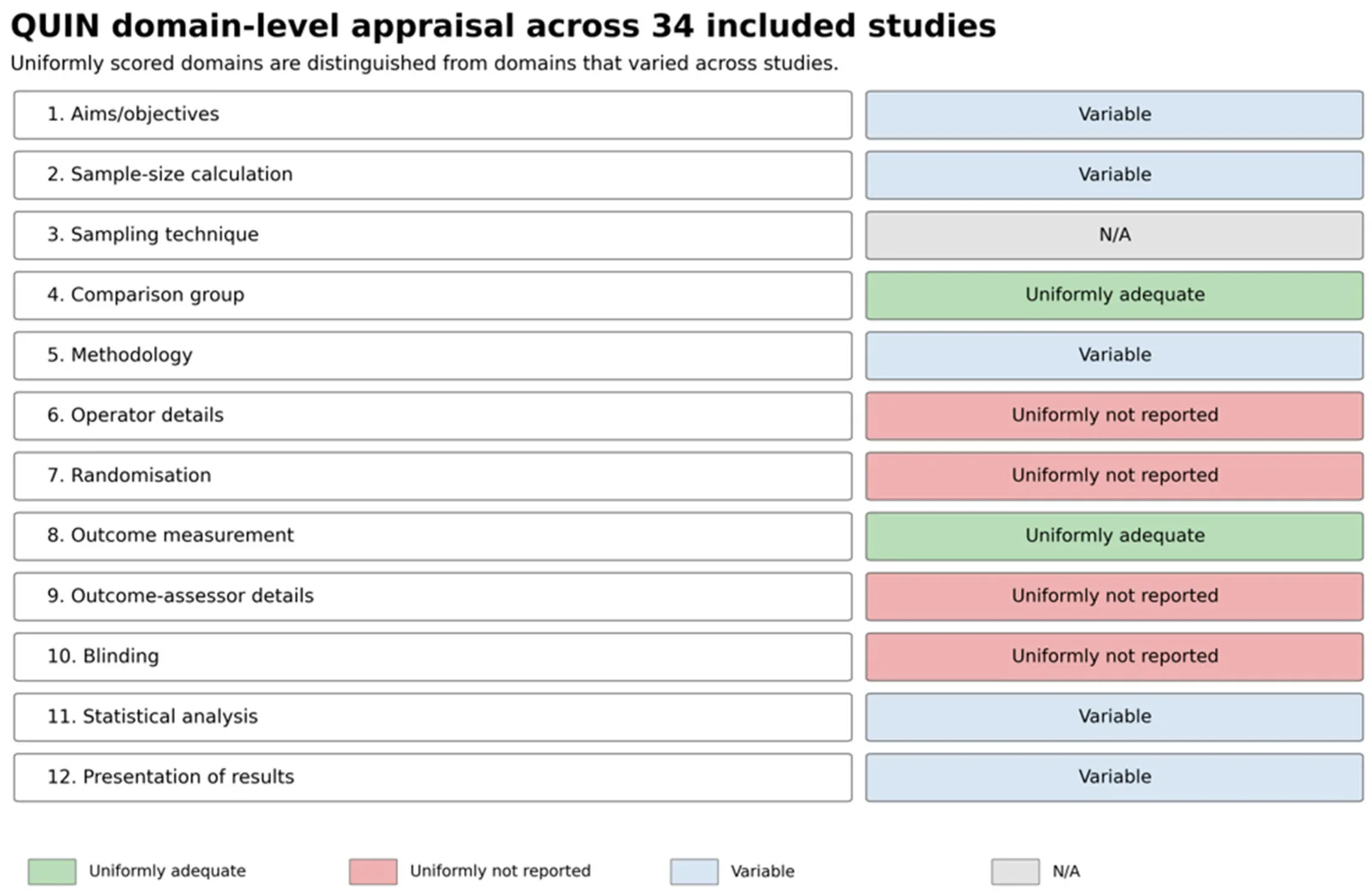
Domain-level summary of the QUIN appraisal of methodological and reporting completeness across the 34 included studies. Four domains were uniformly scored as not reported, two were uniformly scored as adequately addressed, one was uniformly considered not applicable, and five varied across studies. The figure does not display per-study scores.

**Figure 3:**
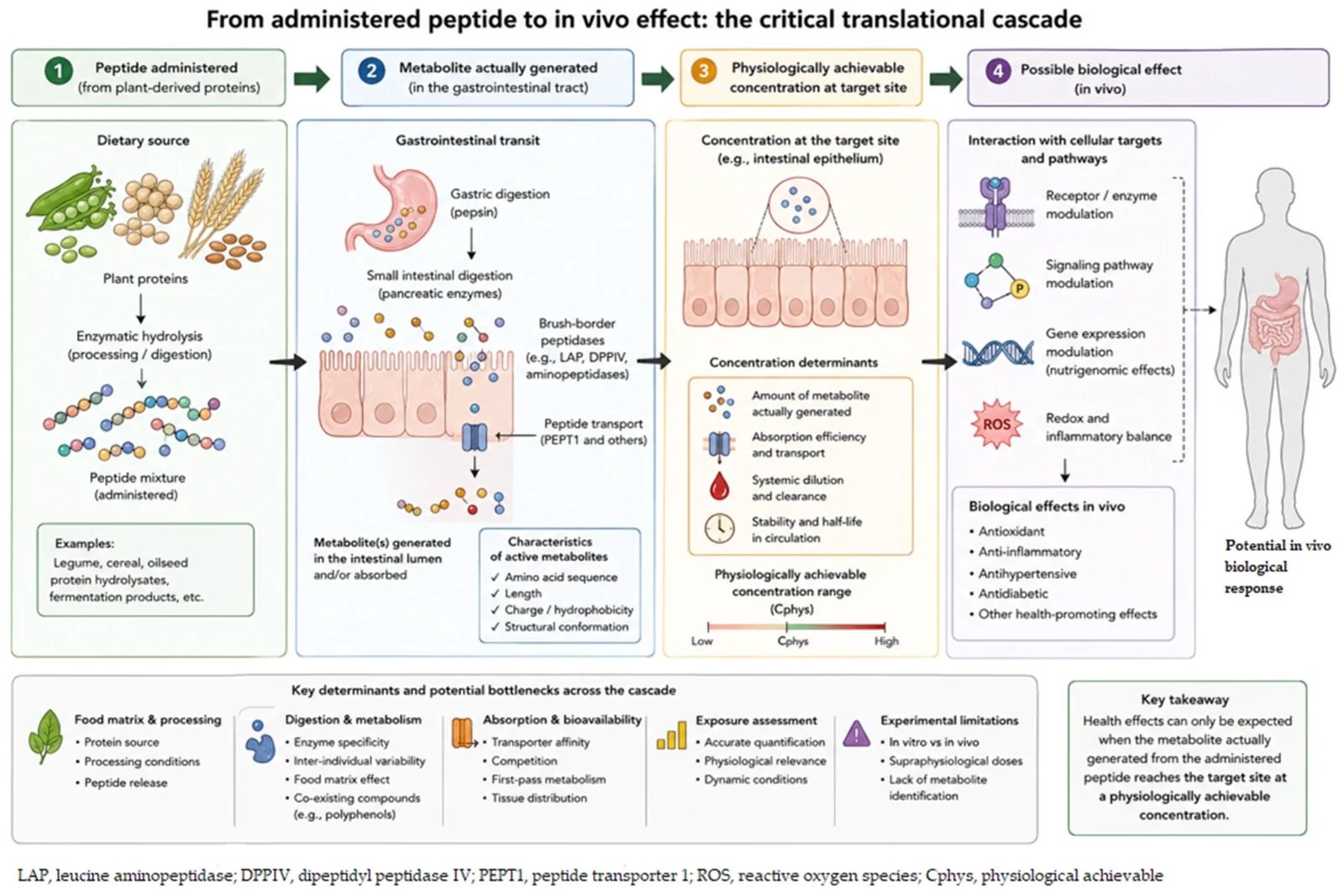
From administered peptide to possible in vivo effect: the critical translational cascade. The biological relevance of a plant-derived peptide depends on sequential demonstration that the administered material generates a defined active metabolite, that this metabolite reaches the intestinal epithelium or another target site at a physiologically achievable concentration, and that target engagement produces a reproducible biological response in vivo. Digestion, brush-border metabolism, absorption, systemic dilution, clearance, and food-matrix effects represent major translational bottlenecks.

Results of individual studies are tabulated by functional domain in Table 2, Table 3, Table 4, Table 5, Table 6 and Table 7. The characterisation status of the bioactive species is tabulated in Table 1, and the tested concentration ranges are compared against an exploratory luminal benchmark in Table 8; the risk-of-bias appraisal is displayed in Figure 2 and the translational cascade in Figure 3.

A quantitative meta-analysis was not performed and was judged not to be feasible. The included studies differ in plant source and food matrix, in the form of the tested material, in the units used to express concentration, in exposure duration, in the presence and nature of the applied stressor, and in the assays used to measure nominally equivalent outcomes; no common comparator is available across studies, and effect estimates with measures of precision were not reported by the primary studies. The evidence was therefore synthesised narratively by direction of effect. For the same reason, statistical exploration of heterogeneity by subgroup analysis or meta-regression was not possible, and sensitivity analyses were not applicable. Possible sources of heterogeneity were instead examined qualitatively, in terms of whether the molecular species responsible for the observed effect had been characterised (Section 3.8) and of whether the tested concentrations were plausibly attainable at the intestinal epithelium (Section 3.9).

The synthesis is accordingly a vote count by direction of effect, the approach recommended for situations of this kind in Chapter 12 of the Cochrane Handbook [46]. Its limitations are explicit and consequential, and should be kept in view when reading Table 2, Table 3, Table 4, Table 5, Table 6 and Table 7. Vote counting takes no account of the magnitude of an effect, so that a 5% and a 500% change in a marker count equally; it takes no account of the variance of the estimate or of the number of independent biological replicates, so that a result obtained in triplicate counts as heavily as one obtained in six independent experiments; and it takes no account of statistical power. A further distortion is specific to this literature: studies employing large factorial designs contribute many rows to the summary tables and can therefore appear visually dominant relative to studies reporting a single condition. To limit this, every count reported in the text is computed at the level of the study and never at the level of the experimental condition, so that each study contributes exactly one vote per outcome domain in which it reports a result, irrespective of how many concentrations, exposure times or fractions it tested. The tables retain the condition-level detail, but no numerical claim made in the text is derived from row counts.

### 2.4. Assessment of Reporting Bias and of Certainty of the Evidence

Risk of bias arising from missing results was not assessed formally, because funnel-plot and regression-based methods require pooled effect estimates that were not computed. The possibility that positive findings are preferentially reported in the in vitro bioactive-peptide literature cannot be excluded and is considered qualitatively in the interpretation of the evidence.

The GRADE approach was not applied. GRADE was developed to rate certainty in bodies of evidence bearing on health outcomes in humans, and no validated adaptation exists for mechanistic cell-culture evidence; applying it here would convey a false impression of comparability with clinical evidence syntheses. Confidence in the body of evidence is therefore conveyed through three explicit elements: the QUIN appraisal of methodological and reporting completeness (Section 2.2), the assessment of whether the molecular species responsible for each reported effect has been identified (Section 3.8), and the assessment of whether the concentrations tested are plausibly attainable in vivo (Section 3.9).

## 3. Results and Discussion

### 3.1. Risk of Bias

The methodological quality of the 34 included publications listed in alphabetical order [12,13,14,15,16,17,18,19,20,21,22,23,24,25,26,27,28,29,30,31,32,33,34,35,36,37,38,39,40,41,42,43,44,45] is shown in Figure 2 and in detail in Supplementary Figure S1. Based on the authors’ QUIN scoring, all publications had a medium risk of bias, with scores ranging from 50% to 63.6%.

A methodological clarification is warranted before presenting this assessment. The tool applied throughout this review, QUIN, was developed for in vitro studies in dentistry and is used here, as in the original assessment, for lack of a validated risk-of-bias instrument specific to nutritional cell-culture research. It is not the Cochrane Risk of Bias 2 (RoB2) tool: RoB2 is designed for randomised controlled trials and evaluates domains such as the randomisation process, deviations from intended interventions, and blinding of trial participants and personnel, none of which apply to a Caco-2 monolayer experiment. Applying RoB2 terminology to this body of literature would misrepresent the study design; the figure therefore presents the QUIN domains actually scored for each study, in the traffic-light format popularised by RoB2 (and by the robvis tool), which we consider the most transparent way to display this information rather than a claim that RoB2 itself was applied.

Four of the twelve QUIN domains take the identical value for every one of the 34 studies: operator details, randomisation, outcome-assessor details, and blinding are scored 0 (not addressed) throughout, while method of measurement of outcome and details of comparison group are scored 2 (adequately addressed) throughout, and sampling technique is uniformly not applicable. This uniformity is not a sign of consistently high or low quality; it reflects that several QUIN domains describe features of clinical or dental study design (blinding of an operator, randomisation of a comparison arm) that are either strictly inapplicable to a single-cell-line assay or applicable in principle but never reported in the present literature. Only one domain, sampling technique, belongs to the first category. The remaining four were scored 0 rather than not applicable, because randomisation of well or plate allocation, blinding of the analyst reading a plate or quantifying an image, and identification of the operator are all feasible in a Caco-2 experiment; their uniform absence is a property of reporting practice in this field rather than of the assay itself. The scoring convention, and a sensitivity analysis quantifying its effect on the risk-of-bias classification, are set out in Section 2.2. In either case, these domains cannot discriminate between the included studies. Conversely, the five domains that do discriminate between studies, namely clearly stated aims, sample-size justification, detailed explanation of the methodology, statistical analysis and presentation of results, are the ones that actually drive the medium-risk classification reported in Section 3.1, and are where the genuine quality differences between these 34 studies are located.

### 3.2. Antioxidant Effect

The published data on the antioxidant effects of plant-derived peptide supplementation in Caco-2 cells are summarised in Table 2.

The number of available studies is limited to 13 cases [12,19,22,28,35,36,37,39,40,41,43,44,45], which cover a wide diversity of food matrices, proteins, and synthetic sequences. Applying the classification of the tested material defined in Section 2.3, seven of these studies tested at least one purified or synthetic single peptide [19,22,35,36,39,41,45], whereas ten tested a chromatographic or ultrafiltration fraction, a whole hydrolysate or a gastrointestinal digest [12,22,28,35,36,37,40,43,44,45], four studies having tested both forms. In the latter group, the antioxidant activity is a property of a compositionally undefined mixture and cannot be attributed to any identified sequence, a distinction maintained throughout the sections that follow (although, as detailed below, the classification into matrix/protein/sequence categories is not mutually exclusive across all 13 studies, and the reported sub-totals do not sum to 13). More specifically, six studies employed protein hydrolysates from gluten meal [40], canary seed [37], soybean [43,44], walnuts [45], and sunflower [28], two studies used intact pure proteins [12,19], two studies used mixed isolated proteins [22,41], one used a whole food product [36], and, finally, six studies employed synthetic sequences [22,35,36,39,41,45]. Among them, six studies performed an in vitro digestion [12,22,35,36,37,43].

Additionally, considerable heterogeneity was observed in experimental conditions across the included studies. Peptide concentrations spanned a broad range, from 0.01 mg/mL [40] to 2.5 mg/mL [37], while incubation times varied from as short as 60 min [37] to as long as 24 h [44], with a single 48-h exposure reported in study [12]. Of note, exogenous oxidative stress was applied in 9 of the 13 studies, achieved by exposing intestinal cell cultures to hydrogen peroxide (H_2_O_2_) at concentrations between 0.2 mM [22] and 50 mM [12], or to tert-butyl hydroperoxide at concentrations ranging from 0.25 mM [36] to 3 mM [19].

Notably, the 50 mM H_2_O_2_ concentration used in one study [12] is two to three orders of magnitude higher than concentrations conventionally applied in sub-lethal oxidative stress protocols in Caco-2 cells [47,48], a discrepancy that should be carefully weighed before adopting this concentration in subsequent work.

The antioxidant effects of peptide supplementation were evaluated using several outcome measures. Reactive oxygen/nitrogen species levels were assessed in 10 studies [12,19,22,28,36,39,40,43,44,45], while lipid peroxidation products were measured in 5 studies [12,36,39,44,45]. Antioxidant status was examined considering the expression [35,36], activity [12,22,28,36,37,39,44,45], or content [22,28,39] of antioxidant molecules. Finally, Nrf2 protein expression was assessed in three studies [36,39,41].

Despite substantial heterogeneity in experimental design across the included studies, the majority of findings pointed in the same direction, with most studies reporting an antioxidant effect of plant-derived food peptides, although this directional agreement should not be read as mechanistic convergence, for the reasons set out below. This effect appears to be driven primarily by a general reduction in oxidant species and oxidised molecules, largely attributable to enhanced activity and content of antioxidant molecules, in turn mediated by upregulation of Nrf2 expression.

Across the antioxidant studies, the reported effects were directionally consistent, with most describing reduced intracellular ROS levels or increased endogenous antioxidant defences. Nevertheless, the magnitude of these effects, the concentrations at which they emerged, and their dependence on exogenous oxidative stress varied by more than an order of magnitude, and the assays employed (for example, DCFH- and DHR-based probes) detect distinct oxidant species and are not directly comparable. Several peptides, including lunasin [19], sunflower hydrolysate [28] and the gastrointestinal digesta of a fermented-soy fraction [36] were inactive under basal conditions and exerted their effect only under exogenous oxidative challenge, indicating modulation of the stress response rather than a constitutive antioxidant activity. Non-monotonic dose–responses were also observed in some studies [19,22], further indicating that the underlying mechanisms are not simply concentration-dependent.

A distinction should be maintained here between what these experiments observe and what they demonstrate. A fall in DCFH- or DHR-derived fluorescence, or a rise in the activity of superoxide dismutase or catalase, is an observed biological effect; it is not by itself evidence of direct radical scavenging by the peptide. The same readouts are produced by several indirect routes: transcriptional induction of the Nrf2 regulon, chelation of transition metals in the medium, competition of the peptide or of co-extracted phenolics with the probe, a reduced number of viable cells in wells where viability is partially compromised, and altered probe loading or esterase activity. Of the 13 antioxidant studies, only 3 measured Nrf2 protein together with downstream antioxidant enzymes [36,39,41], and only 1 controlled for the non-peptide fraction of the matrix [28]; none demonstrated a direct peptide–radical or peptide–Keap1 interaction in the cellular system in which the effect was measured. The mechanistic account given below is therefore an inference consistent with the data rather than a mechanism established within this body of evidence, and it is appraised as such in Section 3.11.

One sequence included among these studies, the α-gliadin peptide LGQQQPFPPQQPY (also known as P31–43), requires separate consideration. This peptide is a well-characterised innate-immune stimulus in coeliac disease, activating IFN-α and TLR7 signalling and interfering with endocytic trafficking in Caco-2 cells, and it prolongs EGFR/ERK signalling [49]. Consistently with this pro-inflammatory role, it reduced antioxidant defences (SOD, GST, GR, GSH/GSSG and Nrf2/Keap1) and increased ROS and MDA levels, behaving as a pro-oxidant stressor rather than as an antioxidant [39]. It is therefore considered here as a negative reference rather than as a candidate functional peptide.

Nrf2 is a master regulator of cellular redox homeostasis, driving the transcription of antioxidant genes in response to oxidative stress. Under basal conditions, Nrf2 is kept in check by Keap1, a cysteine-based redox sensor that targets it for proteasomal degradation via the Cullin-3 ubiquitin ligase complex. Oxidative stimuli disrupt the Keap1/Nrf2 interaction, allowing Nrf2 to translocate to the nucleus and bind ARE/EpRE elements, thereby activating antioxidant gene expression [50]. Given that oxidative stress underlies numerous diseases preventable by food-derived bioactive peptides, it is plausible that these peptides act, at least in part, by modulating Nrf2 activity to enhance cellular antioxidant defences. Since peptides do not appear to act as direct Nrf2 ligands, a key open question concerns the upstream mechanism responsible for the transcriptional responses they induce. Mechanistically, in silico studies suggest that peptides can occupy the active site within the Keap1 Kelch domain, thereby disrupting the Keap1–Nrf2 interaction and impairing Keap1’s association with Cullin-3 [51]. This disruption prevents Nrf2 ubiquitination, leading to its stabilisation, nuclear translocation, and subsequent activation of downstream target genes. At the same time, other peptides, particularly those bearing reactive cysteine residues, may act as covalent modifiers, forming disulfide bonds with the highly reactive cysteine residues of Keap1, potentially inducing a conformational change in the Keap1 homodimer and impairing Cullin-3-dependent ubiquitination of Nrf2 [52].

Concurrently, numerous studies have described bioactive peptides as effective free radical scavengers [53,54,55]. This antioxidant capacity appears to be governed by several structural features, including a low molecular weight, the presence of hydrophobic amino acid residues (His, Trp, Phe, Pro, Gly, Lys, Ile, and Val) bearing indole, imidazole, or pyrrolidine ring systems, as well as the spatial configuration at the N- and C-termini and the identity of amino acids adjacent to key residues. Among these determinants, amino acid composition and sequence exert the greatest influence on peptide antioxidant activity [56].

### 3.3. Anti-Inflammatory Effect

Following application of the selection criteria outlined in the Methods section, five eligible studies investigating the anti-inflammatory properties of plant-derived peptide supplementation in intestinal cell-based models were identified (Table 3) [14,18,30,31,35].

A notable methodological limitation shared across the included studies is the restricted diversity and limited number of protein matrices examined. One study evaluated hemp protein hydrolysates [30], another examined proteins isolated from pea, wheat, potato, and fava bean together with their in vitro gastrointestinal digests [18], a third analysed albumin and globulin fractions from quinoa and amaranth seeds [14], and two further studies tested synthetic peptides derived from gliadin and from the GID of a fermented soy-based beverage [31,35]. Furthermore, experimental conditions varied considerably across studies, with peptide concentrations ranging from 50 µg/mL [30,35] to 5 mg/mL [18], and incubation periods spanning from 30 min [31] to 24 h [18,30,35]. Notably, four studies introduced exogenous inflammatory stimuli [14,18,30,35], exposing cultured intestinal cells to lipopolysaccharide at concentrations ranging from 1 [30] to 20 µg/mL [18], 10 ng/mL interleukin-1β [14], and 2 ng/mL tumour necrosis factor-α [35]. The effects of peptide supplementation on inflammatory responses were assessed through multiple approaches: four studies quantified the mRNA transcript levels of pro-inflammatory [14,30,31,35] or anti-inflammatory [30,31] cytokines; three studies measured the secretion of pro-inflammatory [14,18,30] or anti-inflammatory [30,31] cytokines into the culture medium; and three studies evaluated nuclear factor-κB (NF-κB) protein expression [31,35] or activation [14].

Despite considerable heterogeneity in experimental design, most studies reported suppression of pro-inflammatory cytokines and NF-κB activation following exposure to plant-derived peptides or hydrolysates. This directional agreement should be read alongside two qualifications: first, the gliadin-derived sequence LGQQQPFPPQQPY (α-gliadin P31–43), included among these studies, is a well-characterised pro-inflammatory innate-immune stimulus [57] rather than a beneficial peptide, and behaves in the opposite direction to the others; second, at least one study [18] showed that a pro-inflammatory effect attributed to the intact pea and oat protein disappears after simulated digestion, indicating that some of the heterogeneity across this literature reflects differences in digestion state rather than genuine disagreement about peptide activity.

Two further qualifications follow from the classification of the tested material. Of the five studies in this domain, only two assayed a defined synthetic sequence [31,35]; the remaining three tested hemp hydrolysates [30], intact plant proteins together with their digests [18], or albumin and globulin fractions of quinoa, buckwheat and amaranth seeds [14], so that the anti-inflammatory effect is in most cases a property of a compositionally undefined mixture. In addition, reduced cytokine transcript or secretion levels demonstrate that the inflammatory output of the monolayer is lower, not that the peptide engages a defined target. None of the included studies performed IKK activity assays, p65 chromatin-occupancy measurements or receptor-blockade experiments, and only two examined the IkB/NF-kB axis directly [31,35]. Because sub-lethal loss of viability, reduced cell number and interference with immunoassay detection all lower measured cytokine output, the attribution of these effects to NF-kB inhibition remains a plausible inference rather than a demonstrated mechanism (Section 3.11).

Intestinal inflammation stems from an aberrant immune response to microbial and host-derived signals, often triggered by loss of epithelial barrier integrity. Central to this process is the activation of pattern recognition receptors on epithelial and immune cells, converging on NF-κB. In resting cells, NF-κB is retained in the cytoplasm by IκB proteins; receptor engagement activates the IKK complex, promoting IκB degradation and allowing NF-κB to translocate to the nucleus and induce transcription of inflammatory genes. Key targets include pro-inflammatory cytokines (TNF-α, IL-1β, IL-6) and chemokines such as IL-8, which recruit neutrophils and other leukocytes [58]. These cytokines, in turn, amplify NF-κB signalling in neighbouring cells, sustaining a self-perpetuating inflammatory loop and underscoring the therapeutic relevance of NF-κB and its cytokine targets in chronic intestinal inflammation [59]. This amplification loop explains why NF-κB and its cytokine effectors remain key therapeutic targets in chronic intestinal inflammation.

In this regard, one of the most widely proposed mechanisms underlying the anti-inflammatory activity of bioactive peptides involves the inhibition of NF-κB nuclear translocation and activation, thereby suppressing the subsequent release of pro-inflammatory mediators [60]. In this complex yet intriguing scenario, food-derived bioactive peptides may act through several distinct mechanisms, including stabilisation of IκB to prevent its degradation [35], inhibition of the upstream kinase cascade to reduce IKK complex phosphorylation [61], and direct binding to the active p65 subunit, thereby preventing its nuclear translocation [62]. The potency of this bioactivity appears to be strongly influenced by peptide molecular weight, with low-molecular-weight peptides (less than 1 kDa) generally exhibiting greater anti-inflammatory activity, as well as by amino acid composition, since most peptides displaying anti-inflammatory effects are enriched in hydrophobic and positively charged residues [60].

### 3.4. Antihypertensive Effect

A total of three studies evaluating the antihypertensive effects of plant-derived peptide supplementation in intestinal cell models were identified (Table 4) [13,17,23].

A primary limitation across these investigations is the narrow diversity and limited number of the evaluated matrices. Specifically, one study utilised soybean and pea protein hydrolysates (both ultrafiltered at less than 3 kDa and unfractionated) [13], whereas the remaining two focused on purified peptide sequences derived from lupin [23] and soybean [17]. Furthermore, the experimental conditions varied widely, with peptide concentrations ranging from 10^−7^ M [17,23] to 5 mg/mL [13], and incubation periods spanning from 6 [13] to 24 h [17,23]. Notably, none of the studies introduced exogenous stress stimuli, and ACE activity was employed as the sole biomarker for antihypertensive activity [13,17,23].

ACE functions as a zinc- and chloride-dependent dipeptidase that critically modulates fluid and electrolyte homeostasis, blood pressure regulation, cardiovascular development, and vascular remodelling. These physiological actions are primarily mediated via the enzymatic hydrolysis of angiotensin I into angiotensin II, a potent vasopressor peptide [63]. Although the highest ACE expression has been found in the lungs [64], ACE activity has also been demonstrated in organs like the kidney, heart, intestine, brain, and endocrine organs [63]. To synthesise the vast literature on food ACE-inhibitory peptides, the correlation between their physicochemical and structural properties and ACE inhibitory activity has been recently reviewed [65]. Mechanistically, potent bioactivity is strongly associated with C- or N-terminal hydrophobic clusters that dock into the hydrophobic pockets of the ACE binding site. Proline residues further optimise this interaction by the inherent rigidity of the proline pyrrolidine ring, which stabilises the bioactive peptide conformation, thereby increasing binding affinity. Additionally, charged residues dictate electrostatic complementarity between the peptide and the enzyme. These diverse structural features reflect the complex nature of ACE–peptide interactions, highlighting the importance of motif identification in predicting peptide efficacy.

All three studies reported a reduction of ACE activity in Caco-2 cells. This apparent agreement, however, reflects two mechanistically distinct scenarios, and in two of the three cases the supplemented peptide is not the species directly responsible for the observed inhibition [17,23]. Molecular-modelling analyses reported within these studies indicate that LPYP interacts favourably with both catalytic domains of ACE (HINT scores 2570 and 2830), consistent with competitive inhibition and with its low IC_50_ (5.0 µM) [17]. By contrast, IAVPTGVA does not fit either catalytic site (HINT scores 20 and −374) [17], and LTFPGSAED shows strongly unfavourable scores (−932 and −1810) [23].

The cellular IC50 values reported for these two peptides (14.7 and 13.7 µM, respectively) therefore reflect the activity of brush-border-generated metabolites rather than of the supplemented sequences. IAVPTGVA is cleaved by apical peptidases into AVPTGVA, IAVPT and IAVP, the last of which shows high affinity for both ACE domains (HINT 1956 and 2164) [17], whereas LTFPGSAED is converted by DPP-IV into LTFPG (HINT 975 and 426), a fragment whose antihypertensive activity has been confirmed in spontaneously hypertensive rats [23].

These observations indicate that, in peptidase-competent cellular assays, the bioactive species may be a metabolite generated at the epithelial surface rather than the peptide added to the medium, so that structure–activity relationships based on the nominal sequence should be interpreted with caution. One additional consideration concerns the cell model and endpoints since one study employed undifferentiated Caco-2 cells [17], which exhibit an apical peptidase profile different from mature enterocytes, and all assessed only ACE enzymatic activity without measuring angiotensin II generation or downstream signalling.

### 3.5. Antidiabetic Effect

Table 5 summarises the data published on the antidiabetic effects of plant-based peptide supplementation in Caco-2 cells. The number of available studies is limited to seven cases [13,15,16,21,24,25,42].

Four studies listed above employed plant protein hydrolysates including germinated quinoa [42], soybean [13,24], pea [13] and lupin [16], whereas the remaining three focused on purified peptide sequences derived from lupin [25], soy [25], and malt [21], or on in silico gastrointestinal digestion of garlic [15]. Moreover, experimental conditions varied significantly across the literature. Applied peptide concentrations ranged from 10^−6^ M [25] to 5 mg/mL [13,24], and exposure durations lasted from 1 h [16,25,42] to 24 h [24]. Notably, no exogenous stressors were introduced, leaving DPP-IV activity [13,15,16,24,25,42] and protein expression [21] as the sole biomarker employed to quantify the antidiabetic effect.

Despite substantial heterogeneity in experimental designs across laboratories, most studies reported reduced DPP-IV activity following exposure to plant-derived peptides. However, this directional agreement coexists with limited characterisation of which molecular species is responsible for the effect in most of these studies.

A comparable caution applies to the interpretation of the readout itself. DPP-IV activity was assayed in intact monolayers in every case, so that a decrease in measured activity is equally compatible with competitive inhibition of the ectoenzyme, with allosteric inhibition, with a reduced abundance of DPP-IV at the apical membrane, and with hydrolysis of the peptide by DPP-IV itself acting as an alternative substrate. Only one study distinguished between these possibilities by measuring DPP-IV protein content [21], and none measured incretin concentrations, which the Caco-2 model cannot generate in the absence of enteroendocrine cells (Section 3.11 and Section 3.12).

DPP-IV, a crucial 110 kDa serine protease glycoprotein, occurs as both a membrane-anchored and a soluble isoform. Both variants contribute to the modulation of glucose homeostasis by enzymatically inactivating incretin hormones, including glucagon-like peptide-1 and glucose-dependent insulinotropic polypeptide [66]. Owing to the pivotal role of incretins in mediating postprandial insulin release, the targeted pharmacological inhibition of DPP-IV constitutes a highly promising therapeutic approach for type 2 diabetes management [67].

Bioactive peptides inhibit DPP-IV through multifaceted mechanisms. Beyond conventional competitive binding, where hydrophobic amino acid residues at the C-terminus competitively inhibit DPP-IV activity, forming hydrogen bonds, attractive charge interactions, and hydrophobic interactions at the active site [42], inhibition can occur via allosteric modulation. Specifically, peptide interactions can induce conformational changes that disrupt critical α-helices, leading to structural inactivation [68]. Furthermore, such interactions can trigger cavity shrinkage, reducing the volume of both the active site and the substrate channel, thereby sterically hindering enzymatic activity. Among the DPP-IV inhibitory peptides reported to date in the scientific literature, IPI and VPL have been identified as being among the most potent sequences. Furthermore, the structural motif Xaa1-Pro-Xaa, in which Xaa denotes a hydrophobic amino acid residue, appears to represent a key determinant in modulating the inhibitory potency of DPP-IV-targeting peptides [69]. In addition to direct peptide-mediated inhibition of DPP-IV activity, a nutrigenomic contribution was demonstrated, reflected in the reduction of DPP-IV protein expression levels [21].

### 3.6. Other Biological Effects

A total of four studies investigated additional biological effects of plant-derived peptide supplementation in intestinal cell models (Table 6) [21,26,31,34].

One study employed a lupin protein hydrolysate [26], while the remaining three used isolated sequences from gliadin [31], malt hordein [21] and a hydrolysate from germinated red beans [34]. Tested concentrations ranged from 50 µg/mL [31] to 1 mg/mL [26], with supplementation periods spanning from 30 min [31] to 24 h [26,34]. None of the studies applied an exogenous stressor, leaving the modulation and phosphorylation of selected protein targets as the sole biomarker of the observed biological effects, namely cholesterol metabolism [26], cell signalling [21,31], and global protein expression profiles [34].

Study [26] additionally reported experiments performed in a Caco-2/HepG2 co-culture. Consistent with exclusion criterion (iv) as reformulated in Section 2.1, only the data generated in the Caco-2 monoculture arm of that study were extracted and are reported in Table 6; the co-culture results are neither tabulated nor used to support any conclusion of this review. The same rule was applied to studies [20,27], which combined transport or permeability measurements with biological-activity endpoints: only the viability and bioactivity data were extracted, whereas the transport and apparent-permeability data, which fall under exclusion criterion (iii), were not.

Bioactive peptides may modulate the activity of several transcription factors through the activation of various kinases and phosphatases, triggered by binding to specific plasma membrane receptors, including G protein-coupled receptors, tyrosine-kinase receptors, and integrins [70,71]. This binding activates downstream signal transduction cascades such as MAPK/ERK, PI3K/Akt, and PKC [72,73,74], which in turn phosphorylate target proteins. In this complex and multifaceted scenario, PKA, which can be activated by bioactive peptides [75], may phosphorylate Sterol Regulatory Element-Binding Protein-1a (SREBP-1a), thereby regulating the transcriptional activity of genes involved in cholesterol metabolism [76].

### 3.7. Effects on Cell Viability and Apoptosis

Table 7 summarises the currently available literature on the effect of plant-derived peptides on Caco-2 cell viability and apoptosis.

To date, 22 studies have addressed this topic, encompassing a broad range of food matrices, source proteins, and synthetic or isolated peptide sequences [13,15,16,19,20,22,27,28,29,30,31,32,33,36,37,38,39,40,41,42,43,44]. More specifically, 12 studies employed protein hydrolysates from hemp [30], mustard [33], quinoa [42], gluten meal [40], canary seed [37], soybean [13,43,44], pea [13], lupin [16], sunflower [28], fermented soy beverage [36] and zein [27]. Differently, four studies used intact proteins from kiwicha [38], lunasin [19], wheat [41], and millet [22]. Last, 10 studies employed isolated sequences from wheat [39,41], millet [22], fermented soy beverage [36], garlic [15], walnut [29], gliadin [31,39], beans [20], and *Salvia hispanica* [32]. Among them, seven studies performed an in vitro digestion [15,22,27,36,37,38,43]. The experimental conditions varied widely among the selected studies, with a range of concentrations tested spanning from 10 µg/mL [30,33,40,41] to 50 mg/mL [27] and incubation periods ranging from 1 h [42] to 48 h [13,29]. Among these, oxidative stress was experimentally induced in six studies [19,22,28,36,40,44], whereas the majority of the investigations focused on assessing the impact of peptide supplementation on cell viability [13,15,16,19,20,22,27,28,29,30,31,32,33,36,37,38,39,40,41,42,43,44], with only one study addressing its effect on cell mortality [39] and one addressing apoptosis [32].

Overall, the findings indicate a heterogeneous effect of plant-derived food peptides on Caco-2 cell survival. Specifically, 7 studies reported reduced cell viability or increased mortality [20,28,29,33,38,39,42], 7 studies observed enhanced cell viability [19,22,27,36,40,41,44], and 17 studies found no significant effect on cell viability or mortality [13,15,16,19,20,22,28,30,31,33,36,37,38,39,41,43,44]. In addition, one study reported an increase in apoptotic activity [32].

The interpretation of reduced Caco-2 viability is not uniform across the included studies. Caco-2 is a colorectal adenocarcinoma cell line, and in at least one study the concentration-dependent reduction of viability was regarded as a desirable antiproliferative effect [29], whereas in most others viability assays served only to identify the highest non-cytotoxic concentration for subsequent functional testing. These two interpretations are reported separately, since the same measurement carries an opposite meaning depending on the intended endpoint.

Another case of ambiguity is compounded by the narrow margin between the concentrations eliciting bioactivity and those reducing viability. Where the functional readout is cytokine secretion or ROS generation, a partial loss of viable cells may itself contribute to the observed effect. For this reason, bioactivity reported at concentrations approaching the cytotoxic threshold, and without viability controls at each concentration tested, should be interpreted with caution. Studies aiming to characterise cytoprotective activity in healthy epithelium would benefit from non-transformed models, whereas anticarcinogenic claims require a normal-epithelium comparator to demonstrate selectivity.

In addition, the complexity of interpreting the overall effects of food bioactive peptides on cell viability, and of drawing definitive conclusions, may arise from the wide range of experimental conditions used across studies, including the nature of the sample, the duration of supplementation, peptide concentration, the presence of exogenous stressors, and the assay employed to assess cell viability.

Mechanistically, cytotoxic peptides are generally short, typically containing fewer than 50 amino acids, and are characterised by a net positive charge, marked amphipathicity, the presence of aromatic residues, and defined structural motifs [77]. These features enable them to preferentially recognise, bind to, and disrupt the negatively charged membranes of cancer cells or pathogens through strong electrostatic interactions, while largely sparing healthy cells [78]. Conversely, bioactive peptides may exert a positive effect on cell viability, primarily by acting as antioxidants or by promoting cell proliferation through the modulation of signalling pathways such as PI3K/Akt, MAPK/ERK, and Wnt/β-catenin [79].

### 3.8. Cross-Study Synthesis: Is the Tested Sequence the Bioactive Species?

A recurring finding of this appraisal is that, in a peptidase-competent cellular system, the compound responsible for a measured effect need not be the peptide added to the medium. Table 1 summarises, for all 34 included studies, whether the metabolic fate of the tested material was characterised in the same system in which its bioactivity was measured.

Applying the seven mutually exclusive categories defined in Section 2.3 to the 34 included studies gives the following distribution, in which each study is assigned to one category only, and the percentages are calculated on 34 and sum to 100%. Three studies (9%) identified and independently tested a specific breakdown metabolite (category A) [17,23,25]. Fourteen (41%) tested an uncharacterised fraction or hydrolysate rather than a single sequence, so that the active component or components remain formally unidentified (category B) [13,16,24,26,27,30,33,37,38,40,42,43,44,45]. Six (18%) tested a single purified or synthetic peptide directly, with no assessment of whether it is degraded, and by what, in the assay system (category C) [20,21,29,32,34,41]. Seven (21%) applied a simulated gastrointestinal digestion but did not characterise brush-border-generated products (category D) [12,15,19,22,28,35,36]. Two (6%) tested a sequence already characterised in the independent literature as resistant to intestinal digestion (category E) [31,39]. One (3%) tested an intact parent protein as the primary material (category F) [14]. One (3%) directly compared an intact protein with its gastrointestinal digest (category G), namely study [18], which showed that the reported activity disappears upon digestion; this is the kind of control that, on this evidence, is needed far more often than it is performed.

This distribution indicates that structure–activity claims in this literature rest, for most studies, on the assumption that the administered sequence is the active one, an assumption directly falsified for two of the three antihypertensive studies. The available data do not permit estimation of how often similar conversion occurs in other domains and Table 1 is therefore intended to prioritise confirmatory metabolic-fate studies rather than to imply that the remaining effects are artefactual.

### 3.9. Translational Dose Plausibility by Domain

Table 8 provides an exploratory comparison between concentrations used in Caco-2 experiments and a broad luminal benchmark. A meal containing 20–30 g of plant protein dispersed in approximately 1.5 L of small-intestinal fluid corresponds to about 13–20 mg/mL of total protein-derived material. If an aggregate candidate bioactive-peptide pool represented roughly 1–5% of that material, its concentration would be approximately 0.13–1.0 mg/mL. We therefore use 0.1–1 mg/mL as an order-of-magnitude screening benchmark for mass-based mixtures, not as a sequence-specific exposure estimate. The actual concentration of any single peptide is likely to be substantially lower and depends on digestion yield, food matrix, transit, brush-border metabolism, absorption, and clearance. Molar-only experiments are not converted to mass units unless molecular-weight data are explicitly available.

Mass-based concentrations did not uniformly exceed the exploratory benchmark, but several upper-range experiments did. The largest divergence occurred in the viability literature, where 50 mg/mL was tested, approximately 500-fold above the lower 0.1 mg/mL benchmark. Ratios in Table 8 are screening comparisons rather than pharmacokinetic estimates and do not apply to molar-only studies. In vivo confirmation within an intact organism was available for only one identified metabolite, LTFPG. The translational sequence required before an in vitro response can be interpreted as a plausible dietary effect is summarised in Figure 3.

### 3.10. Structure–Activity Determinants: Sequence, Molecular Weight and Physicochemical Features

Because the biological activity of a peptide is a property of its primary sequence and of the physicochemical features that follow from it, the evidence assembled here is interpretable only if those determinants are made explicit. Three of them recur across all four functional domains. Molecular weight is the first: fractions below 3 kDa were consistently more active than the parent hydrolysates from which they were obtained [40,44,45], and the most potent defined sequences in this corpus are short, ranging from the γ-glutamyl dipeptide γ-EV [20] and the pentapeptide YPQPQ [21] to nonapeptides such as LTFPGSAED [23,25]. Low molecular weight favours access to membrane-proximal and intracellular targets, limits steric hindrance at enzyme active sites, and increases the probability of reaching the epithelium with the active motif intact. Amino acid composition is the second: hydrophobic and aromatic residues (Trp, Tyr, Phe, Pro, His, Ile, Val, Leu) dominate the sequences reported as active here, whether the readout is radical scavenging, ACE inhibition or DPP-IV inhibition, their side chains providing both the electron- and hydrogen-donating capacity relevant to redox activity and the hydrophobic contacts relevant to enzyme binding. Terminal position is the third: activity is disproportionately determined by the residues at the N- and C-termini and by their immediate neighbours, which is precisely why removal of four C-terminal residues from LTFPGSAED converts an ACE-inactive parent into the ACE-active fragment LTFPG [23].

**Figure 4:**
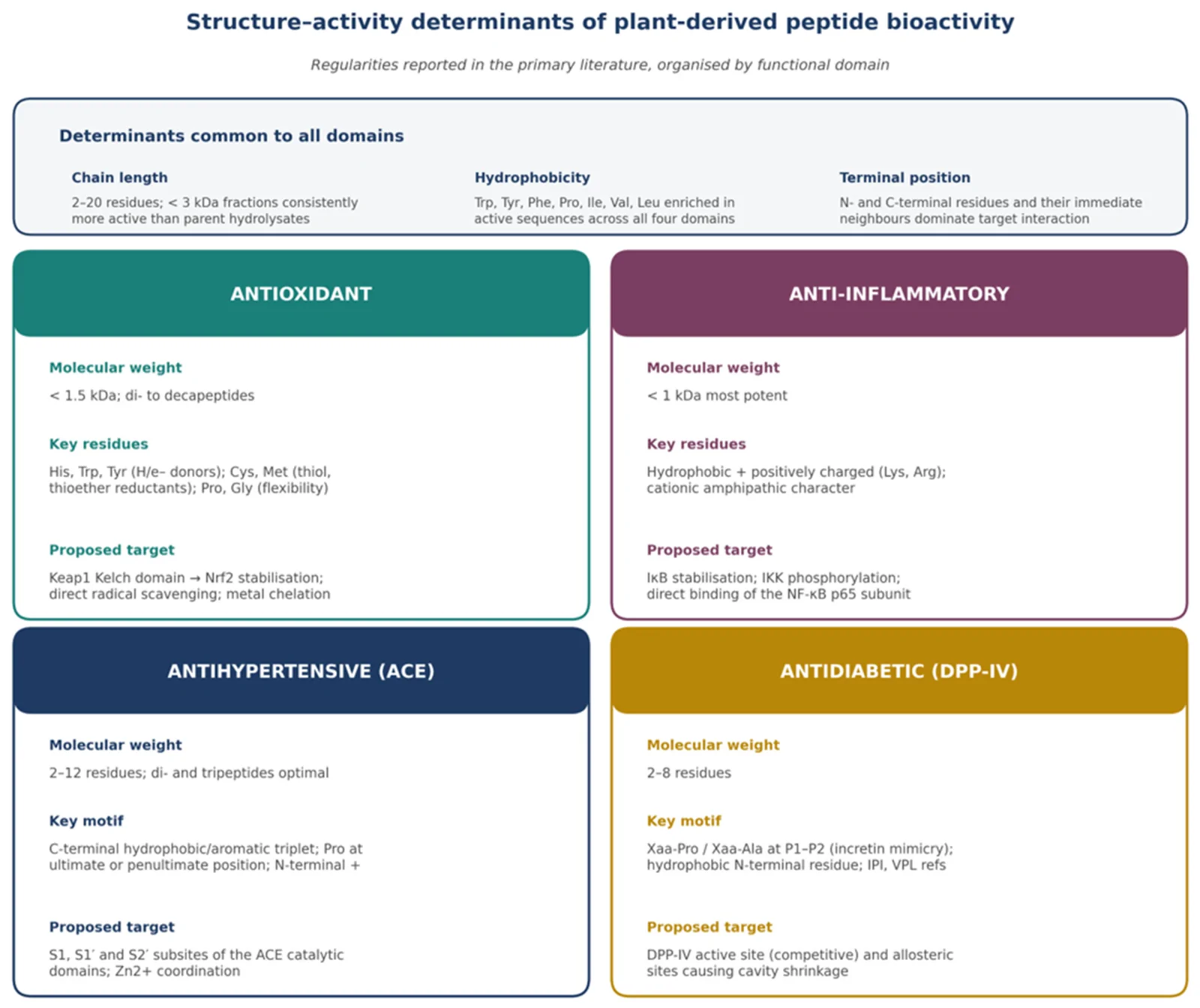
Structure–activity determinants of plant-derived peptide bioactivity by functional domain. For each domain, the panel reports the molecular-weight range associated with activity, the amino acid residues and structural motifs most frequently implicated, and the molecular target engaged; determinants shown in the upper band apply across all domains. The figure summarises regularities reported in the primary literature and does not imply that these relationships were experimentally established within the Caco-2 studies included here (Section 3.10).

These determinants operate differently in each domain, and Figure 4 summarises them. Antioxidant activity is associated with sequences below approximately 1.5 kDa containing His, Trp, Tyr, Cys or Met, in which imidazole, indole and phenolic side chains act as hydrogen or electron donors and thiol and thioether groups as direct reductants, with Pro and Gly contributing the conformational flexibility that exposes them [56]. Anti-inflammatory activity is associated with peptides below approximately 1 kDa enriched in hydrophobic and positively charged residues, a cationic amphipathic composition compatible with membrane association and with interaction with the IKK complex or the p65 subunit [60]. ACE inhibition is associated with a C-terminal hydrophobic or aromatic triplet, with Pro at the ultimate or penultimate position and with a positive charge at the N-terminus, features matching the S1, S1′ and S2′ subsites of the enzyme [65]; the HINT scores reported within the included studies illustrate this directly, since LPYP docks favourably into both catalytic domains, whereas the acidic C-terminus of LTFPGSAED does not [17,23]. DPP-IV inhibition is associated with an Xaa-Pro or Xaa-Ala motif at positions P1–P2, which mimics the natural incretin substrate, together with a hydrophobic N-terminal residue; IPI and VPL remain the reference sequences [69].

Two constraints limit what can legitimately be concluded from these regularities within the present corpus. First, in the 14 studies assigned to category B, the tested material is a mixture whose sequence composition was not determined, or was determined only in part, so that no structure–activity inference is possible: an association between activity and, for example, a sub-3 kDa cut-off is an association with a size class, not with a sequence. Second, in the six studies of category C, the sequence assayed is indeed the sequence supplemented, but its integrity in the assay system was never verified, so an apparent structure–activity relationship may in fact describe a hydrolysis product. Structure–activity conclusions in this field are therefore secure only for the three studies of category A, and it is these that demonstrate the principle most clearly, since in two of them the active structure differs from the administered one [17,23,25].

### 3.11. From Observed Effects to Demonstrated Mechanisms

A recurrent interpretive step in this literature converts an observed change in a biomarker into a statement about mechanism. The distinction matters because almost every endpoint used here is downstream, integrative and open to several independent causes. A decrease in DCFH- or DHR-derived fluorescence indicates that less probe was oxidised in the well: this is compatible with direct scavenging by the peptide, with induction of endogenous antioxidant enzymes, with metal chelation in the medium, with quenching by co-extracted phenolics, with reduced esterase-dependent probe loading, and simply with fewer viable cells. A decrease in secreted IL-8 or TNF-α indicates that less cytokine was present in the medium: this is compatible with inhibition of NF-κB translocation, with reduced transcription through unrelated pathways, with increased cytokine degradation, with interference in the immunoassay, and again with reduced cell number. A decrease in cell-associated ACE or DPP-IV activity indicates that less substrate was converted: this is compatible with competitive inhibition, with allosteric inhibition, with reduced enzyme abundance at the apical membrane, and with the peptide acting as an alternative substrate.

**Figure 5:**
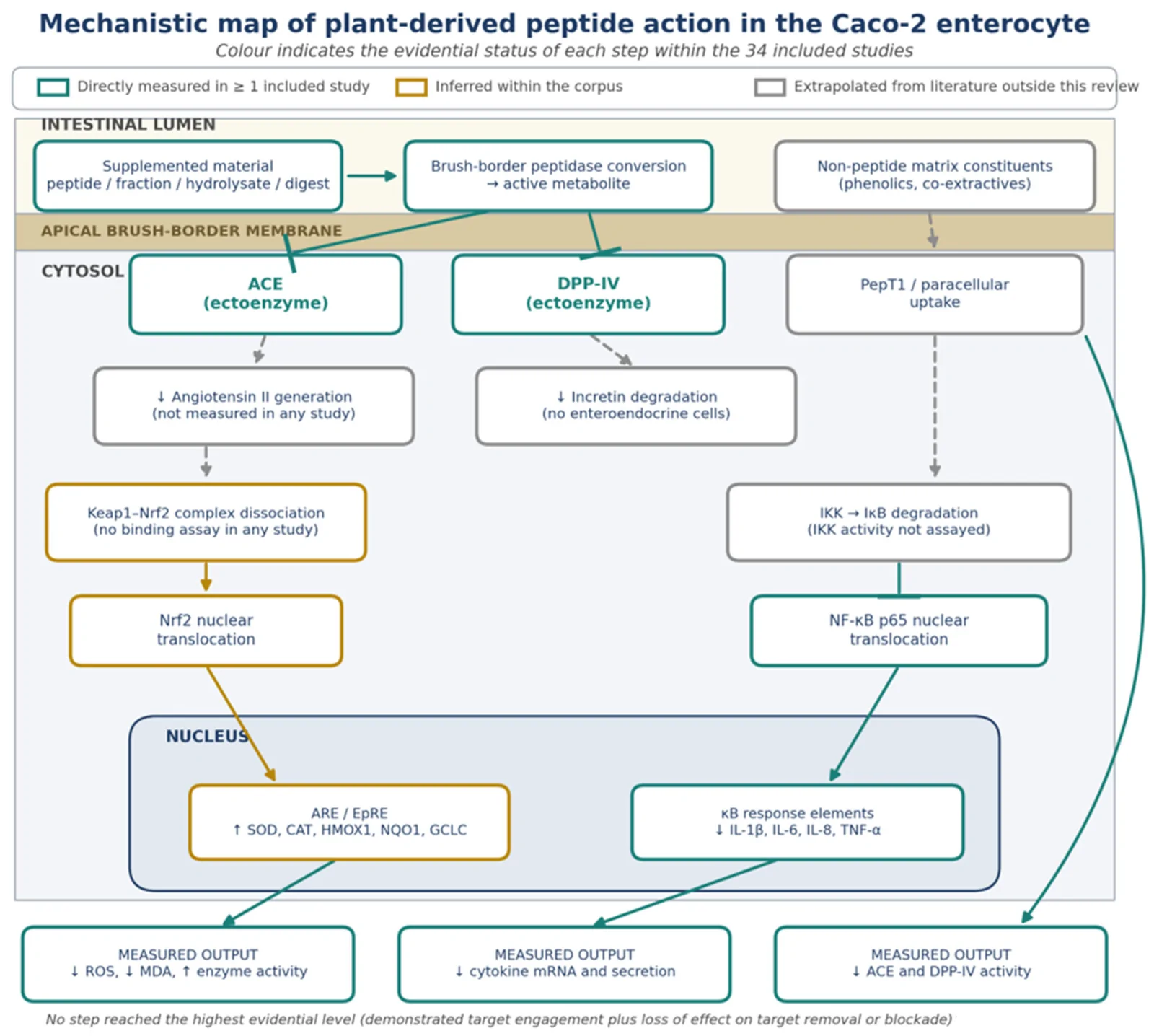
Mechanistic map of plant-derived peptide action in the Caco-2 enterocyte, annotated by evidential status. Apical events include brush-border peptidase conversion of the supplemented sequence into the species that engages the target, inhibition of membrane-anchored ACE and DPP-IV, and possible uptake. Intracellular events include Keap1–Nrf2 dissociation with ARE-driven transcription of antioxidant genes, and IKK–IκB–NF-κB signalling with transcription of pro-inflammatory cytokines. Colour indicates, for each step, whether it was directly measured in at least one included study, inferred from other measurements within the corpus, or extrapolated from literature outside this review. No step reached the highest level of evidence, defined as demonstrated target engagement combined with loss of effect upon target removal or blockade (see Section 3.11). ↑: increase; ↓: decrease.

Judged against this standard, the included studies establish effects far more often than they establish mechanisms, and Figure 5 maps the principal mechanistic hypotheses invoked in this corpus onto the evidence actually available for each. In the antioxidant domain, Nrf2 protein was measured in three studies [36,39,41] and Keap1 in 1 [39]; no study demonstrated peptide binding to Keap1, peptide–radical adduct formation, or nuclear Nrf2 occupancy of ARE sequences in the same cellular system in which the antioxidant effect was observed, and the docking evidence supporting the Keap1 hypothesis [51] was generated outside this corpus. In the anti-inflammatory domain, the IκB/NF-κB axis was examined directly in two studies [31,35] and IKK activity in none. In the antihypertensive domain, the mechanism is best supported, because molecular modelling and metabolite identification were combined and the active species was isolated and retested [17,23]; this is also, instructively, the domain in which the mechanism initially assumed proved to be wrong. In the antidiabetic domain, only 1 study distinguished enzyme inhibition from reduced enzyme abundance [21].

We therefore separate, throughout this review and in Figure 5, three levels of evidential status: an effect observed under the reported conditions; a mechanism supported by at least one direct measurement of the proposed intermediate in the same system; and a mechanism established by demonstration of target engagement together with loss of effect when the target is removed or blocked. No study in this corpus reaches the third level. The mechanistic accounts given in Section 3.2, Section 3.3, Section 3.4 and Section 3.5, which are drawn largely from the wider peptide literature, are accordingly presented as hypotheses consistent with the observed effects and as a research agenda, and not as conclusions of this systematic review.

### 3.12. Limitations of the Caco-2 Model and Emerging Intestinal Systems

Every conclusion of this review is conditioned by the properties of a single cell line. Caco-2 cells derive from a human colorectal adenocarcinoma and, on spontaneous differentiation, acquire a polarised monolayer phenotype with apical microvilli, tight junctions and a brush-border enzyme complement resembling that of the small intestine. They nevertheless differ from the epithelium they model in ways that bear directly on the endpoints reviewed here. They contain no goblet cells and therefore no mucus layer, so peptides reach the apical membrane without traversing the unstirred mucus barrier that in vivo retards diffusion and binds cationic and hydrophobic sequences. They contain no Paneth, enteroendocrine, tuft or M cells, so incretin secretion, antimicrobial-peptide release and antigen sampling cannot be assessed; the absence of enteroendocrine cells is a particular limitation for the antidiabetic domain, in which DPP-IV inhibition is measured while the GLP-1 whose half-life it would extend is not produced. They contain no immune cells, so anti-inflammatory endpoints capture only the epithelial component of a response that in vivo is dominated by lamina propria macrophages, dendritic cells and T lymphocytes. They are cultured aerobically and without microbiota, so microbial peptide metabolism, short-chain fatty acid production and microbe-derived signalling are all excluded. They grow as a flat monolayer without crypt–villus architecture, without the shear stress of luminal flow and without peristaltic deformation, and their tight junctions are tighter than those of the small intestine, which is why absolute permeability values obtained in this system are not directly transferable. Finally, they are transformed cells with an aneuploid karyotype and an altered metabolic phenotype, features that make the interpretation of viability endpoints intrinsically ambiguous, as discussed in Section 3.7, and produce phenotypic drift with passage number and between laboratories [8].

A set of models addressing these limitations individually is now available and is, in our view, where the next generation of studies in this field should be conducted. Co-culture with the mucus-secreting HT29-MTX line restores an adherent mucus layer and yields permeability and efflux behaviour closer to the human intestine, with the goblet-cell fraction tunable through the seeding schedule [80]; addition of Raji B cells induces an M-cell-like phenotype suitable for antigen and particle sampling [81]. Human intestinal organoids, derived from Lgr5-positive stem cells of biopsy material, reproduce the crypt–villus axis and contain the full complement of differentiated lineages, including goblet and enteroendocrine cells, and are genetically non-transformed and donor-specific [82]; plated as two-dimensional monolayers on permeable supports, they become directly compatible with the apical-treatment designs used throughout the studies reviewed here. Microfluidic gut-on-chip systems add luminal flow, cyclic mechanical strain and, in some configurations, stable anaerobic co-culture with commensal bacteria, and have been shown to recapitulate transcriptional, metabolic and immunological responses that monolayers do not [83,84]. None of these systems replaces Caco-2 monolayers for early screening, where standardisation, cost and throughput remain decisive. The argument is rather that a bioactivity first observed in Caco-2 cells should now be treated as a hypothesis to be confirmed in a mucus-competent, brush-border-competent and preferably non-transformed system before it is described as a property of the human intestinal epithelium [85].

### 3.13. Strengths and Limitations of the Present Review

The principal strengths of this review are the explicit, study-by-study appraisal of whether the molecular species responsible for each reported effect was identified, the systematic comparison of tested concentrations against an exposure benchmark, and the consistent separation of purified sequences from compositionally undefined mixtures. Its limitations are set out below and should be weighed against those strengths.

First, the review protocol was not prospectively registered in PROSPERO, or an equivalent register, and no formal written protocol was produced before screening began. The eligibility criteria, screening rules and extraction and appraisal procedures were agreed between the two reviewers in advance and are reported as Protocol S1, but the absence of a time-stamped registration means that deviations from the intended methods cannot be independently verified, and that post hoc adjustment of criteria cannot be formally excluded. Second, the search string was deliberately generic and employed neither controlled vocabulary nor truncation or synonym expansion; although this maximises sensitivity, for the reasons given in Section 2.1, a record indexed under neither of the two search concepts would not have been retrieved. Third, only PubMed and Scopus were searched: Web of Science, Embase, CAB Abstracts and FSTA were not, and no grey literature, preprint, thesis or conference abstract was sought, so publication bias, which in this field is likely to favour positive findings, can be neither excluded nor formally assessed. Fourth, the synthesis is a vote count by direction of effect, with the consequences set out in Section 2.3: effect magnitude, variance, replicate number and statistical power do not enter the summary, and no meta-analysis, effect-size comparison or between-study heterogeneity statistic is reported. Fifth, the QUIN instrument was developed for in vitro research in dentistry and measures reporting completeness rather than demonstrated bias; four of its twelve domains were uninformative for every included study, so the appraisal effectively discriminates on five domains only. Sixth, GRADE was not applied, for the reasons given in Section 2.4, so no certainty rating accompanies the conclusions. Seventh, the luminal benchmark used in Section 3.9 is an order-of-magnitude construct derived from meal protein content and small-intestinal fluid volume rather than a pharmacokinetic estimate, and it does not apply to studies reporting molar concentrations of compositionally undefined material. Eighth, the evidence base is restricted to a single transformed cell line, with the consequences described in Section 3.12. These four domains were scored as not specified rather than not applicable; because the QUIN denominator counts only applicable criteria, this conservative convention determines the medium-risk classification reported here, and the sensitivity analysis in Section 2.2 quantifies its effect.

### 3.14. Final Considerations

The finding that plant-derived peptides can act at the transcriptional level on Nrf2 and NF-κB opens an as-yet-unexamined question, namely whether these peptides also influence the expression levels of ACE and DPP-IV, rather than solely their enzymatic activity. This possibility is supported by a single study within the reviewed literature [21], in which the malt-hordein-derived peptide YPQPQ was shown to lower DPP-IV protein content in Caco-2 cells, a nutrigenomic outcome that differs mechanistically from straightforward enzyme inhibition. By contrast, ACE expression has not been investigated in any of the available studies. Given that ACE is itself regulated by NF-κB and that angiotensin II acts as a strong NF-κB activator, it is mechanistically reasonable to hypothesise a two-way interaction linking these peptides, the renin–angiotensin system, and inflammatory signalling. However, this relationship has yet to be explored in the Caco-2 model.

The influence of digestion also deserves emphasis. The peptide profile reaching the epithelium depends on the food matrix, on peptide concentration and on transit time, and the in vitro digestion protocol most widely adopted (INFOGEST) terminates at the pancreatic phase and does not include brush-border membrane peptidases [86]. Peptides classified as digestion-stable by this protocol are therefore not necessarily stable at the epithelial surface, a distinction of direct consequence given that, as discussed above, the bioactive species in some studies is generated precisely at this stage. Cooking should likewise be considered, as it modifies protein digestibility and thus the spectrum of peptides ultimately released.

The possible contribution of non-peptide constituents represents a further limitation. Only one study tested a hydrolysate depleted of phenolic compounds [28]. In the remaining studies using phenolic-rich matrices, the reported antioxidant activity cannot be confidently attributed to the peptide fraction alone, since co-extracted polyphenols are plausible and often more potent contributors and may act synergistically with the peptides.

Finally, the choice of cell model conditions the interpretation of viability data. The use of a tumour-derived line for assessing both cytoprotection and anticarcinogenicity is intrinsically ambiguous, and viability assays were in most cases used only to identify the highest non-cytotoxic concentration for subsequent functional determinations rather than as a bioactivity endpoint in their own right.

## 4. Conclusions

Given the multifaceted biological effects of bioactive peptides, one of the major challenges in this field is to move beyond the investigation of single, isolated effects and instead adopt high-throughput technologies capable of unravelling their underlying mechanisms of action. In this regard, omics approaches, particularly multi-omics strategies, may represent a powerful tool for identifying key altered genes, proteins, and metabolites, as well as the metabolic and signalling pathways involved.

These conclusions rest on a qualitative, direction-of-effect synthesis rather than on a quantitative pooling of effect sizes, and should be read together with the limitations set out in Section 3.13. Taken together, the evidence reviewed here establishes that plant-derived peptides are biologically active in Caco-2 cells across several functional domains, but it does not yet establish which molecular species are responsible, at what concentrations these effects would occur in vivo, or whether they persist in non-transformed epithelium. Progress will require that the metabolic fate of the supplemented sequence be characterised in the same system in which its activity is measured, that concentrations be justified against realistic intraluminal exposure, and that untargeted multi-omics approaches be combined with brush-border-competent digestion models and non-transformed epithelial systems, so that the field can move from cataloguing effects to identifying mechanisms.

## Figures and Tables

**Table 1:** Characterisation status of the bioactive species across included studies.

Ref.	Peptides/Matrix (Material Class)	Domain	Bioactive-Species Status (Category)	Note
[12]	β-conglycinin GID [HD]	Antioxidant	Partial (GID applied, no brush-border) (category D)	
[13]	Soybean PH [HD, FR]	Antihypertensive/Antidiabetic	Fraction, not single sequence (category B)	Hydrolysate and <3 kDa sub-fraction tested, not isolated sequences
[14]	Albumins from quinoa seeds [IP]	Anti-inflammatory	Intact protein, not peptide (category F)	Intact parent protein tested as the primary material; retained under the parent-protein comparator provision of the inclusion criteria (Section 2.1). Its effects are not attributed to a peptide
[15]	WPHY, VAPGW, PPSPSY, IASW, WPQY (garlic, in silico GID) [PP]	Antidiabetic	Partial (category D)	Activity retested in early-confluent Caco-2 confirming persistence, but no further breakdown analysis; IC_50_ for WPHY inconsistent with source (163.73 vs. 81.57 µM)
[16]	Lupin PH [HD]	Viability	Fraction, not single sequence (category B)	Whole hydrolysate tested
[17]	IAVPTGVA, LPYP (soy) [PP]	Antihypertensive	Metabolite identified (Soy1) (category A); LPYP intact	Soy1 cleaved to IAVP (ACE-active); LPYP itself binds ACE directly
[18]	Pea protein, intact vs. GID [IP, HD]	Anti-inflammatory	Assessed effect abolished by digestion (category G)	Pro-inflammatory IL-8 effect seen only for intact protein, disappears after simulated digestion
[19]	Lunasin (soy) [PP]	Antioxidant	Partial (category D)	Lunasin is a 43-residue peptide generally reported as digestion-stable; not verified in this system
[20]	γ-EV (beans) [PP]	Viability	Not assessed (category C)	Single small molecule, mechanism of cytotoxicity not linked to a specific breakdown product
[21]	YPQPQ (malt hordein) [PP]	Antidiabetic	Not assessed (category C)	Tested directly at fixed concentration; no breakdown-product analysis
[22]	Millet GID < 3 kDa hydrophobic fraction [FR]	Antioxidant	Partial (GID applied, no brush-border) (category D)	
[23]	LTFPGSAED (lupin) [PP]	Antihypertensive/Antidiabetic	Metabolite identified (category A)	Cleaved to LTFPG by DPP-IV; LTFPG is the ACE-active species (HINT+), parent is not (HINT−)
[24]	Peptic/tryptic soybean PH [HD]	Antidiabetic	Fraction, not single sequence (category B)	
[25]	LTFPGSAED (lupin, dose-range) [PP]	Antidiabetic	Metabolite identified (category A)	Same system as [23]
[26]	Peptic/tryptic lupin PH [HD]	Other (lipid metabolism)	Fraction, not single sequence (category B)	
[27]	Zein GID PH [HD]	Viability	Fraction, not single sequence (category B)	
[28]	Sunflower PH, phenol-depleted, <5 kDa [FR]	Antioxidant	Partial (category D)	Only study using a phenol-depleted control
[29]	CTLEW (walnut) [PP]	Viability (anticancer)	Not assessed (category C)	Selectivity vs. IEC-6 shown, but no metabolic-fate analysis
[30]	Hemp PH (Alcalase, 10/20 min) [HD]	Anti-inflammatory	Fraction, not single sequence (category B)	Alcalase hydrolysis time (10 vs. 20 min) changes cytokine profile; not a single characterised peptide
[31]	LGQQQPFPPQQPY = gliadin P31-43 [PP]	Anti-inflammatory/signalling (pro-inflammatory reference)	Known digestion-resistant (category E)	P31-43 is characterised in the coeliac-disease literature as resistant to intestinal digestion. Included as a negative reference: it is a pro-inflammatory innate-immune stimulus, not a candidate beneficial peptide
[32]	KLKKNL, MLKSKR, KKYRVF [PP] (*Salvia hispanica*)	Viability (cell cycle)	Not assessed (category C)	Synthetic peptides tested directly
[33]	Black mustard grain hydrolysate [HD]	Viability	Fraction, not single sequence (category B)	
[34]	LIIPATSTKFL (red bean) [PP]	Viability (anticancer)/Proteomics	Not assessed (category C)	Direct proteomic profiling at IC_50_; no digestion/metabolism step
[35]	Fermented-soy GID fractions [FR, PP]	Antioxidant	Partial (category D)	Peptides selected by docking then tested directly; no further metabolism assessed
[36]	SLVNNDDRDS, DEQIPSHPPR (fermented soy, 2nd study) [FR, PP]	Antioxidant	Partial (category D)	Peptides selected by docking then tested directly; no further metabolism assessed
[37]	Canary seed GID < 3 kDa PH [FR]	Antioxidant	Fraction, not single sequence (category B)	
[38]	Kiwicha GID hydrolysate [HD]	Antioxidant/Antidiabetic/Viability	Fraction, not single sequence (category B)	13 peptides identified but bioactivity attributed to fraction, not isolated sequence
[39]	LGQQQPFPPQQPY = gliadin P31-43 [PP]	Antioxidant domain (pro-oxidant reference)	Known digestion-resistant (category E)	Same peptide as [31]; pro-oxidant and pro-inflammatory, used here as a negative reference and never counted among the antioxidant peptides
[40]	Corn gluten meal hydrolysate fractions [FR]	Antioxidant	Fraction, not single sequence (category B)	
[41]	GIIQPQQPAQLEVIR (wheat) [PP]	Antioxidant	Not assessed (category C)	Synthetic peptide tested directly; no breakdown-product analysis
[42]	Quinoa GID < 1 kDa fraction [FR]	Antidiabetic	Fraction, not single sequence (category B)	Fraction-level IC_50;_ individual peptides not tested in situ
[43]	Soybean PH [HD]	Antioxidant	Fraction, not single sequence (category B)	
[44]	Soybean PH, <3 kDa fraction [HD, FR]	Antioxidant	Fraction, not single sequence (category B)	
[45]	Walnut (*C. cathayensis*)/pecan PH < 3 kDa [FR]	Antioxidant	Fraction, not single sequence (category B)	

Material class: PP, purified or synthetic single peptide; FR, ultrafiltration or chromatographic fraction; HD, whole protein hydrolysate or simulated gastrointestinal digest; IP, intact parent protein. Characterisation categories A–G are defined in Section 2.3. ACE: angiotensin-converting enzyme; GID: (in vitro) gastrointestinal digested; IL-8: Interleukin-8; PH: protein hydrolysate.

**Table 2:** Summary of the antioxidant effects of plant-based peptides in Caco-2 cells.

Ref	Peptides/Matrix	Concentration and Time of Supplementation	Exogenous Stress	Biological Effects
[19]	Lunasin	0.5, 1, 5, 10, and 25 µM for 24 h	N. p.	↔ ROS level
			3 mM TbOOH for 1.5 h	↓ ROS level by DCFH assay; ↓ ROS level at 0.5, 1, and 25 µM by DHR assay
			4 mM H_2_O_2_ for 1.5 h	↓ ROS level by DCFH assay; ↓ ROS level at 1, 5, 10, and 25 µM by DHR assay
[40]	<1 kDa corn gluten meal hydrolysate	0.01, 0.05, 0.1, 0.5, 1 mg/mL for 8 h	1 mM H_2_O_2_ for 6 h	↓ ROS level at 0.05, 0.1, 0.5, 1 mg/mL
	1–3 kDa corn gluten meal hydrolysate	0.01, 0.05, 0.1, 0.5, 1 mg/mL for 8 h	1 mM H_2_O_2_ for 6 h	↓ ROS level at 0.05, 0.1, 0.5, 1 mg/mL
[44]	Soybean PH	0.25, 0.5, and 1 mg/mL for 24 h	1 mM H_2_O_2_ for 3 h	↓ ROS level
	<3 kDa soybean PH	0.25, 0.5, and 1 mg/mL for 24 h	1 mM H_2_O_2_ for 3 h	↓ ROS level
		0.25, 0.5, 0.75, and 1 mg/mL for 24 h	1 mM H_2_O_2_ for 3 h	↓ MDA level; ↑ GP activity; ↑ CAT activity at 1 mg/mL; ↑ GR activity at 0.75 and 1 mg/mL
	3–5 kDa soybean PH	0.25, 0.5, and 1 mg/mL for 24 h	1 mM H_2_O_2_ for 3 h	↓ ROS level
	5–10 kDa soybean PH	0.25, 0.5, and 1 mg/mL for 24 h	1 mM H_2_O_2_ for 3 h	↓ ROS level
	>10 kDa soybean PH	0.25, 0.5, and 1 mg/mL for 24 h	1 mM H_2_O_2_ for 3 h	↓ ROS level
[37]	<3 kDa GID of canary seed PH	0.31, 0.62, 1.25, and 2.50 mg/mL for 1 h	N. p.	↑ CAA
[12]	GID of β-conglycin	0.06 µg for 48 h	N. p	↔ NO_2_ and TBARS levels; ↔ SOD, CAT, and ALP activities
			50 mM H_2_O_2_ for 30 min	↔ NO_2_ and TBARS levels; ↑ SOD and CAT activities; ↔ ALP activity
	GID of deglycosylated β-conglycin	0.06 µg for 48 h	N. p.	↔ NO_2_ and TBARS levels; ↑ SOD and ALP activities; ↔ CAT activity
			50 mM H_2_O_2_ for 30 min	↑ NO_2_ level; ↓ TBARS level; ↑ CAT and SOD activities; ↔ ALP activity
[28]	<5 kDa sunflower PH with reduced phenolic content	0.15, 0.3, and 0.6 mg/mL for 4 h	N. p.	↔ ROS level
			1 mM H_2_O_2_ for 3 h	↓ ROS level
		0.15 mg/mL for 4 h	N. p.	↓ SOD and CAT activities; ↔ GSH content and NO level
			1 mM H_2_O_2_ for 3 h	↓ CAT activity and GSH content; ↔ SOD activity and NO level
[43]	Soybean PH	0.25, 0.5, and 1 mg/mL for 24 h	N. p.	↓ ROS level
			1 mM H_2_O_2_ for 6 h	↓ ROS level
	In vitro gastric digestion of soybean PH	0.25, 0.5, and 1 mg/mL for 24 h	N. p.	↓ ROS level
			1 mM H_2_O_2_ for 6 h	↓ ROS level
	GID of soybean PH	0.25, 0.5, and 1 mg/mL for 24 h	N. p.	↓ ROS level
			1 mM H_2_O_2_ for 6 h	↓ ROS level
[41]	Wheat peptides	50 µg/mL for 36 h	N. p.	↑ Nrf2 protein expression; ↓ Keap1 protein expression
	GIIQPQQPAQLEVIR from wheat peptides	50 µg/mL for 36 h	N. p.	↑ Nrf2 protein expression; ↓ Keap1 protein expression
	LQPFPQPQLPY from wheat peptides	50 µg/mL for 36 h	N. p.	↑ Nrf2 protein expression; ↔ Keap1 protein expression
	QPPFSQQQQPPFSQQ from wheat peptides	50 µg/mL for 36 h	N. p.	↔ Nrf2 protein expression; ↓ Keap1 protein expression
	GIIQPQQPAQLEAIR from wheat peptides	50 µg/mL for 36 h	N. p.	↔ Nrf2 and Keap1 protein expression
[22]	<3 kDa hydrophobic GID of raw millet protein isolate	25, 50, and 100 µg/mL for 24 h	0.2 mM H_2_O_2_ for 4 h	↓ ROS level; ↑ GSH content at 25 and 50 µL/mL; ↔ SOD activity
	<3 kDa hydrophobic GID of germinated millet protein isolate	25, 50, and 100 µg/mL for 24 h	0.2 mM H_2_O_2_ for 4 h	↑ GSH content; ↓ ROS level; ↑ SOD activity at 25 and 50 µL/mL
	<3 kDa hydrophobic GID of boiled germinated millet protein isolate	25, 50, and 100 µg/mL for 24 h	0.2 mM H_2_O_2_ for 4 h	↓ ROS level; ↑ GSH content at 25 and 100 µL/mL; ↔ SOD activity
	<3 kDa hydrophobic GID of microwaved germinated millet protein isolate	25, 50, and 100 µg/mL for 24 h	0.2 mM H_2_O_2_ for 4 h	↑ GSH content; ↓ ROS level; ↑ SOD activity at 100 µL/mL
	SEDDPFD from <3 kDa hydrophobic GID of boiled germinated millet protein isolate	25, 50, and 100 µg/mL for 24 h	0.2 mM H_2_O_2_ for 4 h	↑ and ↓ ROS level at 25 and 50 µL/mL, respectively; ↑ and ↓ GSH content at 25 and 100 µL/mL, respectively; ↑ SOD activity at 25 and 50 µL/mL
	REEGVLIF from <3 kDa hydrophobic GID of boiled germinated millet protein isolate	25, 50, and 100 µg/mL for 24 h	0.2 mM H_2_O_2_ for 4 h	↓ ROS level at 25 and 100 µL/mL; ↑ GSH content and SOD activity
	EAGNKGTLSF from <3 kDa hydrophobic GID of boiled germinated millet protein isolate	25, 50, and 100 µg/mL for 24 h	0.2 mM H_2_O_2_ for 4 h	↓ and ↑ ROS level at 50 and 100 µL/mL, respectively; ↔ GSH content and SOD activity
	MGPIPSTL from <3 kDa hydrophobic GID of boiled germinated millet protein isolate	25, 50, and 100 µg/mL for 24 h	0.2 mM H_2_O_2_ for 4 h	↓ ROS level at 25 and 100 µL/mL; ↑ GSH content at 50 µL/mL; ↑ SOD activity at 100 µL/mL
	EDDQMDPMAK from <3 kDa hydrophobic GID of boiled germinated millet protein isolate	25, 50, and 100 µg/mL for 24 h	0.2 mM H_2_O_2_ for 4 h	↓ ROS level; ↑ GSH content at 100 µL/mL; ↑ SOD activity
	QNWDFCEAWEPCF from <3 kDa hydrophobic GID of boiled germinated millet protein isolate	25, 50, and 100 µg/mL for 24 h	0.2 mM H_2_O_2_ for 4 h	↓ ROS level; ↑ GSH content at 100 µL/mL; ↑ SOD activity
	MSHRGACGCEK from <3 kDa hydrophobic GID of boiled germinated millet protein isolate	25, 50, and 100 µg/mL for 24 h	0.2 mM H_2_O_2_ for 4 h	↓ ROS level; ↑ GSH content and SOD activity
[36]	RP-HPLC fraction 1 of GID of fermented soy beverage	0.05 mg/mL for 24 h	N. p.	↔ ROS level
			0.25 mM TbOOH for 90 min	↓ ROS level
	RP-HPLC fraction 2 of GID of fermented soy beverage	0.05 mg/mL for 24 h	N. p.	↔ ROS level
			0.25 mM TbOOH for 90 min	↔ ROS level
	RP-HPLC fraction 3 of GID of fermented soy beverage	0.05 mg/mL for 24 h	N. p.	↔ ROS level
			0.25 mM TbOOH for 90 min	↓ ROS level
	RP-HPLC fraction 4 of GID of fermented soy beverage	0.05 mg/mL for 24 h	N. p.	↔ ROS level
			0.25 mM TbOOH for 90 min	↓ ROS level
	RP-HPLC fraction 5 of GID of fermented soy beverage	0.05 mg/mL for 24 h	N. p.	↔ ROS level
			0.25 mM TbOOH for 90 min	↔ ROS level
	NALEPDHRVESEGG from GID fermented soy beverage	0.05 mg/mL for 24 h	N. p.	ROS level, nuclear Nrf2/PCNA protein expression, GR, and TrxR activity
			0.25 mM TbOOH for 90 min	↓ ROS and TBARS levels
	KEQQQEQQQEEQPL from GID fermented soy beverage	0.05 mg/mL for 24 h	N. p.	ROS level, GR, and TrxR activity
			0.25 mM TbOOH for 90 min	↔ ROS level
	HEQKEEHEWHRKEE from GID fermented soy beverage	0.05 mg/mL for 24 h	N. p.	ROS level, nuclear Nrf2/PCNA protein expression, GR, and TrxR activity
			0.25 mM TbOOH for 90 min	↓ ROS level
	MRKPQQEEDDDDE from GID fermented soy beverage	0.05 mg/mL for 24 h	N. p.	ROS level, GR, and TrxR activity
			0.25 mM TbOOH for 90 min	↓ ROS level
	GKHQQEEENEGGSI from GID fermented soy beverage	0.05 mg/mL for 24 h	N. p.	ROS level, nuclear Nrf2/PCNA protein expression, GR, and TrxR activity
			0.25 mM TbOOH for 90 min	↔ ROS level
	DEQIPSHPPR from GID fermented soy beverage	0.05 mg/mL for 24 h	N. p.	↔ ROS level, SOD1/GAPDH, GR/GAPDH protein expression, and GR activity; ↑ nuclear Nrf2/PCNA, TrxR1/GAPDH, NQO1/GAPDH protein expression, and TrxR activity
			0.25 mM TbOOH for 90 min	↓ ROS and TBARS levels
	SLVNNDDRDS from GID fermented soy beverage	0.05 mg/mL for 24 h	N. p.	↔ ROS level, SOD1/GAPDH protein expression, GR and TrxR activities; ↑ nuclear Nrf2/PCNA, TrxR1/GAPDH, GR/GAPDH, and NQO1/GAPDH protein expression
			0.25 mM TbOOH for 90 min	↓ ROS and TBARS levels
	FVDAQPQQKEEG from GID fermented soy beverage	0.05 mg/mL for 24 h	N. p.	↔ ROS level
			0.25 mM TbOOH for 90 min	↔ ROS level
	VNPESQQGSPR from GID fermented soy beverage	0.05 mg/mL for 24 h	N. p.	↔ ROS level, SOD1/GAPDH, TrxR1/GAPDH, GR/GAPDH, NQO1/GAPDH protein expression, and TrxR activity; ↑ nuclear Nrf2/PCNA protein expression and GR activity
			0.25 mM TbOOH for 90 min	↓ ROS and TBARS levels
	IGINAENNQRN from GID fermented soy beverage	0.05 mg/mL for 24 h	N. p.	↔ ROS level, TrxR1/GAPDH, GR/GAPDH, NQO1/GAPDH protein expression, GR and TrxR activities; ↑ nuclear Nrf2/PCNA and SOD1/GAPDH protein expression
			0.25 mM TbOOH for 90 min	↓ ROS and TBARS levels
	FGREEGQQQGEE from GID fermented soy beverage	0.05 mg/mL for 24 h	N. p.	↔ ROS level
			0.25 mM TbOOH for 90 min	↔ ROS level
	YLAGNQEQE from GID fermented soy beverage	0.05 mg/mL for 24 h	N. p.	↔ ROS level, GR, and TrxR activity
			0.25 mM TbOOH for 90 min	↔ ROS level
	FSREEGQQQGEQ from GID fermented soy beverage	0.05 mg/mL for 24 h	N. p.	↔ ROS level
			0.25 mM TbOOH for 90 min	↔ ROS level
[45]	<3 kDa of *C. cathayensis* PH	100 µg/mL for 24 h	2 mM H_2_O_2_ for 2 h	↓ ROS level
	<3 kDa of *J. regia* PH	100 µg/mL for 24 h	2 mM H_2_O_2_ for 2 h	↓ ROS level
	<3 kDa of *C. illinoinensis* PH	100 µg/mL for 24 h	2 mM H_2_O_2_ for 2 h	↓ ROS level
	WDPNY from walnut PH	800 µM for 24 h	2 mM H_2_O_2_ for 2 h	↓ ROS and MDA levels; ↑ CAT and SOD activity
	WDPFE from walnut PH	800 µM for 24 h	2 mM H_2_O_2_ for 2 h	↓ ROS and MDA levels; ↑ SOD activity; ↔ CAT activity
	FDPFE from walnut PH	800 µM for 24 h	2 mM H_2_O_2_ for 2 h	↓ ROS and MDA levels; ↑ CAT and SOD activity
[35]	SLVNNDDRDS from GID of fermented soy beverage	0.05 mg/mL for 24 h	N. p.	↑ mRNA HMOX1/ACTB expression
[39]	LGQQQPFPPQQPY from gliadin	100 µg for 24 h	N. p.	↓ SOD, GST, GP, GR activities, GSH/GSSG content, Nrf2/Keap1, and GCLM/ACTB protein expression; ↑ ROS, MDA level, Keap1/ACTB and GCLC/ACTB protein expression; ↔ CAT activity and Nrf2/ACTB protein expression
	LGQQQPFPPQQPY from gliadin and YDWPGGRN from wheat germ	100 µg for 24 h	N. p.	↓ SOD, GP, GR activities, GSH/GSSG content, and GCLM/ACTB protein expression; ↔ ROS level, GST, and CAT activities; ↑ MDA level, Nrf2/Keap1, Keap1/ACTB, Nrf2/ACTB, and GCLC/ACTB protein expression
	LGQQQPFPPQQPY from gliadin and TGP from wheat germ	100 µg for 24 h	N. p.	↔ ROS level, GST and GP activities, and GSH/GSSG content; ↓ SOD and GR activities, Nrf2/Keap1, and GCLM/ACTB protein expression; ↑ CAT activity, MDA level, Keap1/ACTB, Nrf2/ACTB, and GCLC/ACTB protein expression
	LGQQQPFPPQQPY from gliadin and QPYQQPQ from wheat germ	100 µg for 24 h	N. p.	↓ SOD activity; ↔ ROS level, GST, GP, GR activities, and GSH/GSSG content; ↑ MDA level, CAT activity, Nrf2/Keap2, Keap1/ACTB, Nrf2/ACTB, GCLC/ACTB, and GCLM/ACTB protein expression

Effects are referred to the respective unsupplemented control cells either in basal or stressed conditions. In the presence of more than one concentration/treatment time, only statistically significant changes are reported. When samples/concentrations/times are not reported, the effects are referred to all experimental conditions. ↑: increase; ↓: decrease; ↔: no effects; ACTB: β-actin; ALP: alkaline phosphatase; CAA: cellular antioxidant activity; CAT: catalase; DCFH: 2′, 7′-dichlorofluorescein; DHR: dihydrorhodamine; GAPDH: glyceraldehyde-3-phosphate dehydrogenase; GCLC: glutamate–cysteine ligase catalytic subunit; GCLM: glutamate-cysteine ligase modifier subunit; GID: (in vitro) gastrointestinal digested; GP: glutathione peroxidase; GR: glutathione reductase; GSH: reduced glutathione; GSSG: oxidised glutathione; HMOX1: heme oxygenase-1; Keap1: kelch-like ECH-associated protein 1; MDA: malondialdehyde; N. p.: not present; NO: nitric oxide; NO_2_: nitrogen dioxide; NQO1: NAD(P)H:quinone acceptor oxidoreductase 1; Nrf2: nuclear factor erythroid 2-related factor 2; PH: protein hydrolysate; ROS: reactive oxygen species; RP-HPLC: reversed-phase high-performance liquid chromatography; SOD: superoxide dismutase; TBARS: thiobarbituric acid reactive substances; TbOOH: tert butyl hydroperoxide; TrxR: thioredoxin reductase; TrxR1: thioredoxin reductase 1.

**Table 3:** Summary of the anti-inflammatory effects of plant-based peptides in Caco-2 cells.

Ref	Peptides/Matrix	Concentration and Time of Supplementation	Exogenous Stress	Biological Effects
[30]	Hemp PH with Alcalase for 10 min	50 and 100 μg/mL for 24 h	1 μg/mL LPS for 25 h	↔ mRNA IL-1β expression; ↑ mRNA IL-10 expression at 100 μg/mL; ↓ IL-1β, TNF-α, and IL-6 secretion; ↑ IL-10 and IL-4 secretion
	Hemp PH with Alcalase for 20 min	50 and 100 μg/mL for 24 h	1 μg/mL LPS for 25 h	↔ mRNA IL-1β expression; ↓ mRNA TNFα expression; ↑ mRNA IL-10 and mRNA IL-4 expression at 100 μg/mL; ↑ mRNA IL-13 expression; ↓ IL-1β, TNF-α, and IL-6 secretion; ↑ IL-10 and IL-4 secretion
	Hemp PH with Alcalase for 30 min	50 and 100 μg/mL for 24 h	1 μg/mL LPS for 25 h	↔ mRNA IL-1β expression; ↓ mRNA TNF-α expression at 50 μg/mL; ↑ mRNA IL-13 expression at 100 μg/mL; ↓ IL-1β, TNF-α, and IL-6 secretion; ↑ IL-10 and IL-4 secretion
	Hemp PH with Alcalase for 45 min	50 and 100 μg/mL for 24 h	1 μg/mL LPS for 25 h	↔ mRNA IL-1β expression; ↑ mRNA IL-17 and mRNA IL-13 expression; ↑ mRNA IL-10 expression at 100 μg/mL; ↑ mRNA IL-14 expression at 50 μg/mL; ↓ IL-1β, TNF-α, and IL-6 secretion; ↑ IL-10 and IL-4 secretion
	Hemp PH with Alcalase for 60 min	50 and 100 μg/mL for 24 h	1 μg/mL LPS for 25 h	↔ mRNA IL-1β expression; ↑ mRNA IL-10 and mRNA IL-13 expression at 100 μg/mL; ↓ IL-1β, TNF-α, and IL-6 secretion; ↑ IL-10 and IL-4 secretion
	Hemp PH with Alcalase for 60 min and with Flavourzyme for 15 min	50 and 100 μg/mL for 24 h	1 μg/mL LPS for 25 h	↔ mRNA IL-1β expression; ↑ mRNA IL-10 expression; ↑ mRNA IL-4 and mRNA IL-13 expression at 100 μg/mL; ↓ IL-1β, TNF-α, and IL-6 secretion; ↑ IL-10 and IL-4 secretion
	Hemp PH with Alcalase for 60 min and with Flavourzyme for 30 min	50 and 100 μg/mL for 24 h	1 μg/mL LPS for 25 h	↔ mRNA IL-1β expression; ↑ mRNA TNF-α and mRNA IL-13 expression; ↑ mRNA IL-6 expression at 100 μg/mL; ↓ IL-1β, TNF-α, and IL-6 secretion; ↑ IL-10 and IL-4 secretion
	Hemp PH with Alcalase for 60 min and with Flavourzyme for 60 min	50 and 100 μg/mL for 24 h	1 μg/mL LPS for 25 h	↔ mRNA IL-1β expression; ↑ mRNA TNF-α, mRNA IL-6, and mRNA IL-13 expression; ↓ IL-1β, TNF-α, and IL-6 secretion; ↑ IL-10 and IL-4 secretion
	Hemp PH with Alcalase for 60 min and with Flavourzyme for 90 min	50 and 100 μg/mL for 24 h	1 μg/mL LPS for 25 h	↔ mRNA IL-1β expression; ↑ mRNA TNF-α and mRNA IL-6 expression at 50 μg/mL; ↑ mRNA IL-13 expression; ↓ IL-1β, TNF-α, and IL-6 secretion; ↑ IL-10 secretion at 50 μg/mL; ↑ IL-4 secretion
	Hemp PH with Alcalase for 60 min and with Flavourzyme for 120 min	50 and 100 μg/mL for 24 h	1 μg/mL LPS for 25 h	↔ mRNA IL-1β expression; ↑ mRNA IL-13 expression; ↓ IL-1β, TNF-α, and IL-6 secretion; ↑ IL-10 secretion at 50 μg/mL; ↑ IL-4 secretion
[14]	Albumins from quinoa seeds	1 mg/mL for 4 h	N. p.	↔ NF-κB activation and mRNA IL-8 expression
			10 ng/mL IL-1β for 4 h	↓ NF-κB activation and mRNA IL-8 expression
	Albumins from amaranth seeds	1 mg/mL for 4 h	N. p.	↔ NF-κB activation and mRNA IL-8 expression
			10 ng/mL IL-1β for 4 h	↓ NF-κB activation and mRNA IL-8 expression
	Albumins from buckwheat seeds	1 mg/mL for 4 h	N. p.	↔ NF-κB activation and mRNA IL-8 expression
			10 ng/mL IL-1β for 4 h	↓ NF-κB activation and mRNA IL-8 expression
	VLC globulins from quinoa seeds	1 mg/mL for 4 h	N. p.	↔ NF-κB activation and mRNA IL-8 expression
			10 ng/mL IL-1β for 4 h	↓ NF-κB activation and mRNA IL-8 expression
	VLC globulins from amaranth seeds	1 mg/mL for 4 h	N. p.	↔ NF-κB activation and mRNA IL-8 expression
			10 ng/mL IL-1β for 4 h	↓ NF-κB activation and mRNA IL-8 expression
	VLC globulins from buckwheat seeds	1 mg/mL for 4 h	N. p.	↔ NF-κB activation and mRNA IL-8 expression
			10 ng/mL IL-1β for 4 h	↓ NF-κB activation and mRNA IL-8 expression
	LC globulins from quinoa seeds	1 mg/mL for 4 h	N. p.	↔ NF-κB activation and mRNA IL-8 expression
			10 ng/mL IL-1β for 4 h	↓ NF-κB activation and mRNA IL-8 expression
	LC globulins from amaranth seeds	1 mg/mL for 4 h	N. p.	↔ NF-κB activation and mRNA IL-8 expression
			10 ng/mL IL-1β for 4 h	↓ NF-κB activation and mRNA IL-8 expression
	LC globulins from buckwheat seeds	1 mg/mL for 4 h	N. p.	↔ NF-κB activation and mRNA IL-8 expression
			10 ng/mL IL-1β for 4 h	↓ NF-κB activation and mRNA IL-8 expression
	HC globulins from quinoa seeds	1 mg/mL for 4 h	N. p.	↔ NF-κB activation and mRNA IL-8 expression
			10 ng/mL IL-1β for 4 h	↓ NF-κB activation and mRNA IL-8 expression
	HC globulins from amaranth seeds	1 mg/mL for 4 h	N. p.	↔ NF-κB activation and mRNA IL-8 expression
			10 ng/mL IL-1β for 4 h	↓ NF-κB activation and mRNA IL-8 expression
	HC globulins from buckwheat seeds	1 mg/mL for 4 h	N. p.	↔ NF-κB activation and mRNA IL-8 expression
			10 ng/mL IL-1β for 4 h	↓ NF-κB activation and mRNA IL-8 expression
	GID of albumins from quinoa seeds	1 mg/mL for 4 h	N. p.	↔ NF-κB activation and mRNA IL-8 expression
			10 ng/mL IL-1β for 4 h	↓ NF-κB activation and mRNA IL-8 expression
	GID of albumins from amaranth seeds	1 mg/mL for 4 h	N. p.	↔ NF-κB activation and mRNA IL-8 expression
			10 ng/mL IL-1β for 4 h	↓ NF-κB activation and mRNA IL-8 expression
	GID of albumins from buckwheat seeds	1 mg/mL for 4 h	N. p.	↔ NF-κB activation and mRNA IL-8 expression
			10 ng/mL IL-1β for 4 h	↓ NF-κB activation and mRNA IL-8 expression
	GID of VLC globulins from quinoa seeds	1 mg/mL for 4 h	N. p.	↔ NF-κB activation and mRNA IL-8 expression
			10 ng/mL IL-1β for 4 h	↓ NF-κB activation and mRNA IL-8 expression
	GID of VLC globulins from amaranth seeds	1 mg/mL for 4 h	N. p.	↔ NF-κB activation and mRNA IL-8 expression
			10 ng/mL IL-1β for 4 h	↓ NF-κB activation and mRNA IL-8 expression
	GID of VLC globulins from buckwheat seeds	1 mg/mL for 4 h	N. p.	↔ NF-κB activation and mRNA IL-8 expression
			10 ng/mL IL-1β for 4 h	↓ NF-κB activation and mRNA IL-8 expression
	GID of LC globulins from quinoa seeds	1 mg/mL for 4 h	N. p.	↔ NF-κB activation and mRNA IL-8 expression
			10 ng/mL IL-1β for 4 h	↓ NF-κB activation and mRNA IL-8 expression
	GID of LC globulins from amaranth seeds	1 mg/mL for 4 h	N. p.	↔ NF-κB activation and mRNA IL-8 expression
			10 ng/mL IL-1β for 4 h	↓ NF-κB activation and mRNA IL-8 expression
	GID of LC globulins from buckwheat seeds	1 mg/mL for 4 h	N. p.	↔ NF-κB activation and mRNA IL-8 expression
			10 ng/mL IL-1β for 4 h	↓ NF-κB activation and mRNA IL-8 expression
	GID of HC globulins from quinoa seeds	1 mg/mL for 4 h	N. p.	↔ NF-κB activation and mRNA IL-8 expression
			10 ng/mL IL-1β for 4 h	↓ NF-κB activation and mRNA IL-8 expression
	GID of HC globulins from amaranth seeds	1 mg/mL for 4 h	N. p.	↔ NF-κB activation and mRNA IL-8 expression
			10 ng/mL IL-1β for 4 h	↓ NF-κB activation and mRNA IL-8 expression
	GID of HC globulins from buckwheat seeds	1 mg/mL for 4 h	N. p.	↔ NF-κB activation and mRNA IL-8 expression
			10 ng/mL IL-1β for 4 h	↓ NF-κB activation and mRNA IL-8 expression
[18]	Pea protein—grade 1	0.1, 0.25, and 0.5% (*w*/*v*) for 24 h	N. p.	↑ IL-8 secretion
			20 µg/mL LPS for 24 h	↑ IL-8 secretion
	GID of pea protein—grade 1	0.1, 0.25, and 0.5% (*w*/*v*) for 24 h	N. p.	↔ IL-8 secretion
			20 µg/mL LPS for 24 h	↔ IL-8 secretion
	Pea protein—grade 2	0.1, 0.25, and 0.5% (*w*/*v*) for 24 h	N. p.	↔ IL-8 secretion
			20 µg/mL LPS for 24 h	↔ IL-8 secretion
	GID of pea protein—grade 2	0.1, 0.25, and 0.5% (*w*/*v*) for 24 h	N. p.	↔ IL-8 secretion
			20 µg/mL LPS for 24 h	↔ IL-8 secretion
	Pea protein—grade 3	0.1, 0.25, and 0.5% (*w*/*v*) for 24 h	N. p.	↑ IL-8 secretion
			20 µg/mL LPS for 24 h	↑ IL-8 secretion at 0.25 and 0.5% (*w*/*v*)
	GID of pea protein—grade 3	0.1, 0.25, and 0.5% (*w*/*v*) for 24 h	N. p.	↔ IL-8 secretion
			20 µg/mL LPS for 24 h	↔ IL-8 secretion
	Pea protein—grade 4	0.1, 0.25, and 0.5% (*w*/*v*) for 24 h	N. p.	↑ IL-8 secretion
			20 µg/mL LPS for 24 h	↑ IL-8 secretion
	GID of pea protein—grade 4	0.1, 0.25, and 0.5% (*w*/*v*) for 24 h	N. p.	↔ IL-8 secretion
			20 µg/mL LPS for 24 h	↔ IL-8 secretion
	Pea PH	0.1, 0.25, and 0.5% (*w*/*v*) for 24 h	N. p.	↔ IL-8 secretion
			20 µg/mL LPS for 24 h	↔ IL-8 secretion
	GID of pea PH	0.1, 0.25, and 0.5% (*w*/*v*) for 24 h	N. p.	↑ IL-8 secretion at 0.5% (*w*/*v*)
			20 µg/mL LPS for 24 h	↔ IL-8 secretion
	Wheat protein—grade 1	0.1, 0.25, and 0.5% (*w*/*v*) for 24 h	N. p.	↑ IL-8 secretion at 0.5% (*w*/*v*)
			20 µg/mL LPS for 24 h	↔ IL-8 secretion
	GID of wheat protein—grade 1	0.1, 0.25, and 0.5% (*w*/*v*) for 24 h	N. p.	↔ IL-8 secretion
			20 µg/mL LPS for 24 h	↔ IL-8 secretion
	Wheat protein—grade 2	0.1, 0.25, and 0.5% (*w*/*v*) for 24 h	N. p.	↑ IL-8 secretion at 0.5% (*w*/*v*)
			20 µg/mL LPS for 24 h	↔ IL-8 secretion
	GID of wheat protein—grade 2	0.1, 0.25, and 0.5% (*w*/*v*) for 24 h	N. p.	↑ IL-8 secretion at 0.1 and 0.5% (*w*/*v*)
			20 µg/mL for 24 h	↔ IL-8 secretion
	Potato protein	0.1, 0.25, and 0.5% (*w*/*v*) for 24 h	N. p.	↑ IL-8 secretion at 0.1 and 0.25% (*w*/*v*)
			20 µg/mL LPS for 24 h	↑ IL-8 secretion at 0.25% (*w*/*v*)
	GID of potato protein	0.1, 0.25, and 0.5% (*w*/*v*) for 24 h	N. p.	↑ IL-8 secretion at 0.5% (*w*/*v*)
			20 µg/mL LPS for 24 h	↑ IL-8 secretion at 0.5% (*w*/*v*)
	Fava bean protein	0.1, 0.25, and 0.5% (*w*/*v*) for 24 h	N. p.	↔ IL-8 secretion
			20 µg/mL LPS for 24 h	↔ IL-8 secretion
	GID of fava bean protein	0.1, 0.25, and 0.5% (*w*/*v*) for 24 h	N. p.	↔ IL-8 secretion
			20 µg/mL LPS for 24 h	↔ IL-8 secretion
	Oat protein	0.1, 0.25, and 0.5% (*w*/*v*) for 24 h	N. p.	↑ IL-8 secretion
			20 µg/mL LPS for 24 h	↑ IL-8 secretion
	GID of oat protein	0.1, 0.25, and 0.5% (*w*/*v*) for 24 h	N. p.	↔ IL-8 secretion
			20 µg/mL LPS for 24 h	↔ IL-8 secretion
[31]	LGQQQPFPPQQPY from gliadin	100 µg/mL for 30 min	N. p.	↑ pY-NF-κB/NF-κB protein expression
		100 µg/mL overnight	N. p.	↑ mRNA IFN-α 7 and IFN- α 17 protein expression
[35]	SLVNNDDRDS from GID of fermented soy beverage	0.05 mg/mL for 24 h	N. p.	↔ nuclear NF-κB/PCNA protein expression; ↑ cytosolic IκB/GAPDH protein expression; ↔ mRNA IL-1β/ACTB, IL-6/ACTB, TNF-α/ACTB, and IL-1ra/ACTB expression
			2 ng/mL TNF-α for 2.5 h	↓ nuclear NF-κB/PCNA protein expression; ↑ cytosolic IκB/GAPDH protein expression; ↓ mRNA IL-1β/ACTB and TNF-α/ACTB expression; ↑ mRNA IL-1ra/ACTB expression
			0.01 mg/mL LPS	↑ TNF-α secretion

Effects are referred to the respective unsupplemented control cells either in basal or stressed conditions. In the presence of more than one concentration/treatment time, only statistically significant changes are reported. When samples/concentrations/times are not reported, the effects are referred to all experimental conditions. ↑: increase; ↓: decrease; ↔: no effects; ACTB: β-actin; GAPDH: glyceraldehyde-3-phosphate dehydrogenase; GID: (in vitro) gastrointestinal digested; HC: high charge; IFN-α 7: interferon α 7; IκB: Inhibitor of kappa B; IL-10: interleukin-10; IL-13: interleukin-13; IL-17: interleukin-17; IL-1ra: interleukin-1 receptor antagonist; IL-1β: interleukin 1 beta; IL-4: interleukin-4; IL-6: interleukin 6; LC: low charge; LPS: lipopolysaccharides; N. p.: not present; NF-κB: nuclear factor kappa-light-chain-enhancer of activated B cells; PH: protein hydrolysate; pY-NF-κB: phosphotyrosine nuclear factor kappa-light-chain-enhancer of activated B cells; TNF-α: tumour necrosis factor alpha; VLC: very low charge.

**Table 4:** Summary of the antihypertensive effects of plant-based peptides in Caco-2 cells.

Ref	Peptides/Matrix	Concentration and Time of Supplementation	Exogenous Stress	Biological Effects
[13]	Soybean PH	0.1, 0.5, 1, and 5 mg/mL for 6 h	N. p.	↓ ACE activity
	<3 kDa soybean PH	0.1, 0.5, 1, and 5 mg/mL for 6 h	N. p.	↓ ACE activity at 0.5, 1, and 5 mg/mL
	Pea PH	0.1, 0.5, 1, and 5 mg/mL for 6 h	N. p.	↓ ACE activity
	<3 kDa pea PH	0.1, 0.5, 1, and 5 mg/mL for 6 h	N. p.	↓ ACE activity
[23]	LTFPGSAED from lupin	10^−7^, 10^−6^, 10^−5^, 10^−4^, 10^−3.5^ M for 24 h	N. p.	↓ ACE activity (IC_50_ = 13.7 µM)
[17]	IAVPTGVA from soybean	10^−7^, 10^−6^, 10^−5^, 10^−4^, 10^−3.5^ M for 24 h	N. p.	↓ ACE activity (IC_50_ = 14.7 µM)
	LPYP from soybean	10^−7^, 10^−6^, 10^−5^, 10^−4^, 10^−3.5^ M for 24 h	N. p.	↓ ACE activity (IC_50_ = 5.0 µM)

Effects are referred to the respective unsupplemented control cells either in basal or stressed conditions. In the presence of more than one concentration/treatment time, only statistically significant changes are reported. When samples/concentrations/times are not reported, the effects are referred to all experimental conditions. ↓: decrease; ACE: angiotensin-converting enzyme; N. p.: not present; PH: protein hydrolysate.

**Table 5:** Summary of the antidiabetic effects of plant-based peptides in Caco-2 cells.

Ref	Peptides/Matrix	Concentration and Time of Supplementation	Exogenous Stress	Biological Effects
[42]	<1 kDa germinated quinoa PH	0.5, 1, 2, and 3 mg/mL for 1 h	N. p.	↓ DPP-IV activity (IC_50_ = 2.20 mg/mL)
[13]	Soybean PH	1, 2,5 and 5 mg/mL for 6 h	N. p.	↓ DPP-IV activity
	Pea PH	1, 2,5 and 5 mg/mL for 6 h	N. p.	↓ DPP-IV activity
[24]	Peptic soybean PH	1, 2.5, and 5 mg/mL for 24 h	N. p.	↓ DPP-IV activity
	Tryptic soybean PH	1, 2.5, and 5 mg/mL for 24 h	N. p.	↓ DPP-IV activity
[16]	Lupin PH	0.5, 1, and 5 mg/mL for 1, 3, and 6 h	N. p	↓ DPP-IV activity
[15]	WPHY from in silico GID of garlic	From 0 to 2000 µM for 70 min	N. p.	↓ DPP-IV activity (IC_50_ = 163.73 µM)
	VAPGW from in silico GID of garlic	From 0 to 2400 µM for 70 min	N. p.	↓ DPP-IV activity (IC_50_ = 693.52 µM)
	PPSPSY from in silico GID of garlic	From 0 to 2000 µM for 70 min	N. p.	↓ DPP-IV activity (IC_50_ = 692.60 µM)
	IASW from in silico GID of garlic	From 0 to 2800 µM for 70 min	N. p.	↓ DPP-IV activity (IC_50_ = 441.38 µM)
	WPQY from in silico GID of garlic	From 0 to 2000 µM for 70 min	N. p.	↓ DPP-IV activity (IC_50_ = 644.39 µM)
[25]	LTFPGSAED from lupin	100 µM for 1 h	N. p.	↓ DPP-IV activity
		10^−6^, 10^−5^, 10^−4^, 10^−3.5^, 10^−3^, and 10^−2^ M for 1 h	N. p.	↓ DPP-IV activity (IC_50_ = 207.5 µM)
	IAVPTGVA from soy	100 µM for 1 h	N. p.	↓ DPP-IV activity
		10^−6^, 10^−5^, 10^−4^, 10^−3.5^, 10^−3^, and 10^−2^ M for 1 h	N. p.	↓ DPP-IV activity (IC_50_ = 223.5 µM)
[21]	YPQPQ from malt hordein	23.84 µM for 2 and 4 h	N. p.	↓ DPP-IV/ACTB protein expression

Effects are referred to the respective unsupplemented control cells either in basal or stressed conditions. In the presence of more than one concentration/treatment time, only statistically significant changes are reported. When samples/concentrations/times are not reported, the effects are referred to all experimental conditions. ↓: decrease; ACTB: β-actin; DPP-IV: dipeptidyl peptidase-IV; GID: gastrointestinal digested; N. p.: not present; PH: protein hydrolysate.

**Table 6:** Summary of miscellaneous effects of plant-based peptides in Caco-2 cells.

Ref	Peptides/Matrix	Concentration and Time of Supplementation	Exogenous Stress	Biological Effects
[26]	Peptic lupin PH	1 mg/mL for 24 h	N. p.	↑ SREBP2/ACTB, LDLR/ACTB, HMGCoAR/ACTB, pHMGCoAR/ACTB protein expression; ↔ PCSK9-M/ACTB protein expression; ↑ PCSK9-M secretion
	Tryptic lupin PH	1 mg/mL for 24 h	N. p.	↑ SREBP2/ACTB, LDLR/ACTB, HMGCoAR/ACTB, pHMGCoAR/ACTB protein expression; ↔ mature PCSK9/ACTB protein expression; ↓ mature PCSK9 secretion
[31]	LGQQQPFPPQQPY from gliadin	100 µg/mL for 30 min	N. p.	↑ pY-ERK/ERK protein expression
		50 and 100 µg/mL for 30 min	N. p.	↑ pY-ERK/ERK protein expression
		100 µg/mL for 30 min and 3 h	N. p.	↑ TLR7/MyD88 protein expression
		100 µg/mL for 3 h	N. p.	↑ TLR7/EGFR protein expression
		100 µg/mL for 30 min, 3 and 6 h	N. p.	↑ pY-p38/p38 and MxA/tubulin protein expression; ↑ pY-JNK/JNK and TLR7/tubulin protein expression at 30 min and 3 h; ↑ MyD88/tubulin protein expression at 30 min; ↑ pY-ERK/ERK protein expression at 30 min and 6 h
[34]	LIIPATSTKFL from peptic germinated red bean	490.625 µg/mL for 24 h	N. p.	↑ ANKRD50, KLC4, PIK3AP1, SFN, CDK1, UBE2V1, RPL22, GTF2E2, WDHD1, NAV3, MYL6, HSPA4, PCNXL4, ATRNL1, ANXA3, PXMP4, and ARAP1 protein expression
[21]	YPQPQ from malt hordein	23.84 µM for 2 and 4 h	N. p.	↓ ERK/ACTB and p-ERK/ACTB protein expression

Effects are referred to the respective unsupplemented control cells either in basal or stressed conditions. In the presence of more than one concentration/treatment time, only statistically significant changes are reported. When samples/concentrations/times are not reported, the effects are referred to all experimental conditions. ↑: increase; ↓: decrease; ↔: no effects; ACTB: β-actin; ANKRD50: ankyrin repeat domain-containing protein 50; ANXA3: annexin A3; ARAP1: arf-GAP with Rho-GAP domain, ANK repeat and PH domain-containing protein 1; ATRNL1: attractin-like protein 1; CDK1: cyclin-dependent kinase 1; EGFR: epidermal growth factor receptor; ERK: extracellular signal-regulated kinase; GTF2E2: transcription initiation factor IIE subunit beta; HMGCoAR: 3-hydroxy-3-methylglutaryl co-enzyme A reductase; HSPA4: heat shock 70 kDa protein 4; JNK: cJun NH(2)-terminal kinase; KLC4: kinesin light chain 4; LDLR: low density lipoprotein receptor; MxA: myxovirus resistance protein A; MyD88: myeloid differentiation primary response 88; MYL6: myosin light polypeptide 6; N. p.: not present; NAV3: neuron navigator 3; PCNXL4: pecanex-like protein 4; PCSK9: proprotein Convertase Subtilisin/Kexin type 9; PH: protein hydrolysate; pHMGCoA: phosphorylated 3-hydroxy-3-methylglutaryl co-enzyme A reductase; PIK3AP1: phosphoinositide 3-kinase adapter protein 1; PXMP4: peroxisomal membrane protein 4; p-ERK: phosphor: extracellular signal-regulated kinase; pY-ERK: phosphotyrosine extracellular signal-regulated kinase; pY-JNK: phosphotyrosine cJun NH(2)-terminal kinase; pY-p38: phosphotyrosine p38; RPL22: large ribosomal subunit protein eL22; SFN: 14-3-3 protein sigma; TLR7: toll-like receptor 7; UBE2V1: ubiquitin-conjugating enzyme E2 variant 1; WDHD1: WD repeat and HMG-box DNA-binding protein 1.

**Table 7:** Summary of effects on cell survival of plant-based peptides in Caco-2 cells.

Ref	Peptides/Matrix	Concentration and Time of Supplementation	Exogenous Stress	Biological Effects
[30]	Hemp PH with Alcalase for 10 min	10, 50, 100, and 200 μg/mL for 24 h	N. p.	↔ cell viability
	Hemp PH with Alcalase for 20 min	10, 50, 100, and 200 μg/mL for 24 h	N. p.	↔ cell viability
	Hemp PH with Alcalase for 30 min	10, 50, 100, and 200 μg/mL for 24 h	N. p.	↔ cell viability
	Hemp PH with Alcalase for 45 min	10, 50, 100, and 200 μg/mL for 24 h	N. p.	↔ cell viability
	Hemp PH with Alcalase for 60 min	10, 50, 100, and 200 μg/mL for 24 h	N. p.	↔ cell viability
	Hemp PH with Alcalase for 60 min and with Flavourzyme for 15 min	10, 50, 100, and 200 μg/mL for 24 h	N. p.	↔ cell viability
	Hemp PH with Alcalase for 60 min and with Flavourzyme for 30 min	10, 50, 100, and 200 μg/mL for 24 h	N. p.	↔ cell viability
	Hemp PH with Alcalase for 60 min and with Flavourzyme for 60 min	10, 50, 100, and 200 μg/mL for 24 h	N. p.	↔ cell viability
	Hemp PH with Alcalase for 60 min and with Flavourzyme for 90 min	10, 50, 100, and 200 μg/mL for 24 h	N. p.	↔ cell viability
	Hemp PH with Alcalase for 60 min and with Flavourzyme for 120 min	10, 50, 100, and 200 μg/mL for 24 h	N. p.	↔ cell viability
[33]	Black mustard grain hydrolysate	From 0.01 to 10 mg/mL for 24 h	N. p.	↔ cell viability
	Germinated black mustard grain hydrolysate	From 0.01 to 10 mg/mL for 24 h	N. p.	↓ cell viability (IC_50_ = 2.85 mg/mL)
	White mustard grain hydrolysate	From 0.01 to 10 mg/mL for 24 h	N. p.	↓ cell viability (IC_50_ = 7.99 mg/mL)
	Germinated white mustard grain hydrolysate	From 0.01 to 10 mg/mL for 24 h	N. p.	↓ cell viability (IC_50_ = 1.22 mg/mL)
[42]	<1 kDa germinated quinoa PH	0.1, 0.2, 0.4, 0.8, 1.5, 2, 3, and 4 mg/mL for 1 h	N. p.	↓ cell viability at 4 mg/mL
[40]	<1 kDa corn gluten meal hydrolysate	0.01, 0.05, 0.1, 0.5, 1 mg/mL for 8 h	1 mM H_2_O_2_ for 6 h	↑ cell viability at 0.05, 0.1, 0.5, 1 mg/mL
	1–3 kDa corn gluten meal hydrolysate	0.01, 0.05, 0.1, 0.5, 1 mg/mL for 8 h	1 mM H_2_O_2_ for 6 h	↑ cell viability at 0.05, 0.1, 0.5, 1 mg/mL
[37]	<3 kDa GID of canary seed PH	0.31, 0.62, 1.25, and 2.50 mg/mL for 24 h	N. p.	↔ cell viability
[13]	Soybean PH	1, 2,5 and 5 mg/mL for 48 h	N. p.	↔ cell viability
	Pea PH	1, 2,5 and 5 mg/mL for 48 h	N. p.	↔ cell viability
[44]	Soybean PH	0.25, 0.5, and 1 mg/mL for 24 h	N. p.	↔ cell viability
			1 mM H_2_O_2_ for 3 h	↑ cell viability
	<3 kDa soybean PH	0.25, 0.5, and 1 mg/mL for 24 h	N. p.	↔ cell viability
			1 mM H_2_O_2_ for 3 h	↑ cell viability
	3–5 kDa soybean PH	0.25, 0.5, and 1 mg/mL for 24 h	N. p.	↔ cell viability
			1 mM H_2_O_2_ for 3 h	↑ cell viability;
	5–10 kDa soybean PH	0.25, 0.5, and 1 mg/mL for 24 h	N. p.	↔ cell viability
			1 mM H_2_O_2_ for 3 h	↑ cell viability;
	>10 kDa soybean PH	0.25, 0.5, and 1 mg/mL for 24 h	N. p.	↔ cell viability
			1 mM H_2_O_2_ for 3 h	↑ cell viability at 0.5 and 1 mg/mL
[27]	GID of zein PH	5, 10, 25, and 50 mg/mL for 24 h	N. p.	↑ cell viability at 5 and 10 mg/mL
[38]	Kiwicha protein concentrate	From 0.05 to 4 mg/mL for 24 h	N. p.	↔ cell viability
	Gastric digestion of kiwicha protein concentrate	From 0.05 to 4 mg/mL for 24 h	N. p.	↓ cell viability (IC_50_ = 0.72 mg/mL)
	Mid-term GID of kiwicha protein concentrate	From 0.05 to 4 mg/mL for 24 h	N. p.	↓ cell viability (IC_50_ = 0.08 mg/mL)
	>5 kDa mid-term GID of kiwicha protein concentrate	From 0.05 to 4 mg/mL for 24 h	N. p.	↓ cell viability (IC_50_ = 0.33 mg/mL)
	<5 kDa mid-term GID of kiwicha protein concentrate	From 0.05 to 2.5 mg/mL for 24 h	N. p.	↔ cell viability
	GID of kiwicha protein concentrate	From 0.05 to 4 mg/mL for 24 h	N. p.	↓ cell viability (IC_50_ = 0.14 mg/mL)
	>5 kDa GID of kiwicha protein concentrate	From 0.05 to 4 mg/mL for 24 h	N. p.	↓ cell viability (IC_50_ = 0.19 mg/mL)
	<5 kDa GID of kiwicha protein concentrate	From 0.05 to 4 mg/mL for 24 h	N. p.	↓ cell viability (IC_50_ = 1.76 mg/mL)
	RP-HPLC fraction 1 of mid-term GID of kiwicha protein concentrate	From 0.05 to 4 mg/mL for 24 h	N. p.	↓ cell viability (IC_50_ = 1.17 mg/mL)
	RP-HPLC fraction 2 of mid-term GID of kiwicha protein concentrate	From 0.05 to 4 mg/mL for 24 h	N. p.	↓ cell viability (IC_50_ = 0.87 mg/mL)
[16]	Lupin PH	0.1, 0.5, and 5 mg/mL for 48 h	N. p.	↔ cell viability
[28]	<5 kDa sunflower PH with reduced phenolic content	0.15, 0.3, and 0.6 mg/mL for 4 h	N. p.	↓ cell viability at 0.3 and 0.6 mg/mL
			1 mM H_2_O_2_ for 3 h	↔ cell viability
[19]	Lunasin	0.5, 1, 5, 10, and 25 µM for 24 h	N. p.	↔ cell viability
			3 mM TbOOH for 1.5 h	↑ cell viability at 1, 10, and 25 µM
			4 mM H_2_O_2_ for 1.5 h	↑ cell viability at 1 µM
[43]	Soybean PH	0.25, 0.5, and 1 mg/mL for 24 h	N. p.	↔ cell viability
	In vitro gastric digestion of soybean PH	0.25, 0.5, and 1 mg/mL for 24 h	N. p.	↔ cell viability
	GID of soybean PH	0.25, 0.5, and 1 mg/mL for 24 h	N. p.	↔ cell viability
[41]	Wheat peptides	10, 25, 50, 100, 200, 400, and 800 µg/mL for 36 h	N. p.	↑ cell viability at 50 µg/mL
	GIIQPQQPAQLEVIR from wheat peptides	50 and 100 µg/mL for 36 h	N. p.	↔ cell viability
	LQPFPQPQLPY from wheat peptides	50 and 100 µg/mL for 36 h	N. p.	↔ cell viability
	QPPFSQQQQPPFSQQ from wheat peptides	50 and 100 µg/mL for 36 h	N. p.	↔ cell viability
	GIIQPQQPAQLEAIR from wheat peptides	50 and 100 µg/mL for 36 h	N. p.	↔ cell viability
[22]	<3 kDa hydrophobic GID of raw millet protein isolate	25, 50, and 100 µg/mL for 24 h	0.2 mM H_2_O_2_ for 4 h	↑ cell viability
	<3 kDa hydrophobic GID of germinated millet protein isolate	25, 50, and 100 µg/mL for 24 h	0.2 mM H_2_O_2_ for 4 h	↑ cell viability
	<3 kDa hydrophobic GID of boiled germinated millet protein isolate	25, 50, and 100 µg/mL for 24 h	0.2 mM H_2_O_2_ for 4 h	↑ cell viability
	<3 kDa hydrophobic GID of microwaved germinated millet protein isolate	25, 50, and 100 µg/mL for 24 h	0.2 mM H_2_O_2_ for 4 h	↑ cell viability
	SEDDPFD from <3 kDa hydrophobic GID of boiled germinated millet protein isolate	25, 50, and 100 µg/mL for 24 h	N. p.	↔ cell viability
	REEGVLIF from <3 kDa hydrophobic GID of boiled germinated millet protein isolate	25, 50, and 100 µg/mL for 24 h	N. p.	↔ cell viability
	EAGNKGTLSF from <3 kDa hydrophobic GID of boiled germinated millet protein isolate	25, 50, and 100 µg/mL for 24 h	N. p.	↔ cell viability
	MGPIPSTL from <3 kDa hydrophobic GID of boiled germinated millet protein isolate	25, 50, and 100 µg/mL for 24 h	N. p.	↔ cell viability
	EDDQMDPMAK from <3 kDa hydrophobic GID of boiled germinated millet protein isolate	25, 50, and 100 µg/mL for 24 h	N. p.	↔ cell viability
	QNWDFCEAWEPCF from <3 kDa hydrophobic GID of boiled germinated millet protein isolate	25, 50, and 100 µg/mL for 24 h	N. p.	↔ cell viability
	MSHRGACGCEK from <3 kDa hydrophobic GID of boiled germinated millet protein isolate	25, 50, and 100 µg/mL for 24 h	N. p.	↔ cell viability
[36]	SPE fraction of GID of fermented soy beverage	0.05 mg/mL for 24 h	N. p.	↔ cell viability
			0.2 mM TbOOH for 18 h	↑ cell viability
	RP-HPLC fraction 1 of GID of fermented soy beverage	0.05 mg/mL for 24 h	N. p.	↑ cell viability
			0.2 mM TbOOH for 18 h	↑ cell viability
	RP-HPLC fraction 2 of GID of fermented soy beverage	0.05 mg/mL for 24 h	N. p.	↑ cell viability
			0.2 mM TbOOH for 18 h	↑ cell viability
	RP-HPLC fraction 3 of GID of fermented soy beverage	0.05 mg/mL for 24 h	N. p.	↑ cell viability
			0.2 mM TbOOH for 18 h	↑ cell viability
	RP-HPLC fraction 4 of GID of fermented soy beverage	0.05 mg/mL for 24 h	N. p.	↑ cell viability
			0.2 mM TbOOH for 18 h	↑ cell viability
	RP-HPLC fraction 5 of GID of fermented soy beverage	0.05 mg/mL for 24 h	N. p.	↑ cell viability
			0.2 mM TbOOH for 18 h	↑ cell viability
	NALEPDHRVESEGG from GID fermented soy beverage	0.05 mg/mL for 24 h	N. p.	↔ cell viability
			0.2 mM TbOOH for 18 h	↑ cell viability
	KEQQQEQQQEEQPL from GID fermented soy beverage	0.05 mg/mL for 24 h	N. p.	↔ cell viability
			0.2 mM TbOOH for 18 h	↔ cell viability
	HEQKEEHEWHRKEE from GID fermented soy beverage	0.05 mg/mL for 24 h	N. p.	↔ cell viability
			0.2 mM TbOOH for 18 h	↔ cell viability
	MRKPQQEEDDDDE from GID fermented soy beverage	0.05 mg/mL for 24 h	N. p.	↔ cell viability
			0.2 mM TbOOH for 18 h	↑ cell viability
	GKHQQEEENEGGSI from GID fermented soy beverage	0.05 mg/mL for 24 h	N. p.	↔ cell viability
			0.2 mM TbOOH for 18 h	↑ cell viability
	DEQIPSHPPR from GID fermented soy beverage	0.05 mg/mL for 24 h	N. p.	↔ cell viability
			0.2 mM TbOOH for 18 h	↑ cell viability
	SLVNNDDRDS from GID fermented soy beverage	0.05 mg/mL for 24 h	N. p.	↔ cell viability
			0.2 mM TbOOH for 18 h	↑ cell viability
	FVDAQPQQKEEG from GID fermented soy beverage	0.05 mg/mL for 24 h	N. p.	↔ cell viability
			0.2 mM TbOOH for 18 h	↑ cell viability
	VNPESQQGSPR from GID fermented soy beverage	0.05 mg/mL for 24 h	N. p.	↔ cell viability
			0.2 mM TbOOH for 18 h	↑ cell viability
	IGINAENNQRN from GID fermented soy beverage	0.05 mg/mL for 24 h	N. p.	↔ cell viability
			0.2 mM TbOOH for 18 h	↑ cell viability
	FGREEGQQQGEE from GID fermented soy beverage	0.05 mg/mL for 24 h	N. p.	↔ cell viability
			0.2 mM TbOOH for 18 h	↔ cell viability
	YLAGNQEQE from GID fermented soy beverage	0.05 mg/mL for 24 h	N. p.	↔ cell viability
			0.2 mM TbOOH for 18 h	↑ cell viability
	FSREEGQQQGEQ from GID of fermented soy beverage	0.05 mg/mL for 24 h	N. p.	↔ cell viability
			0.2 mM TbOOH for 18 h	↔ cell viability
[15]	WPHY from in silico GID of garlic	150, 300, 600, 1200, and 2400 µM for 24 h	N. p.	↔ cell viability
	VAPGW from in silico GID of garlic	150, 300, 600, 1200, and 2400 µM for 24 h	N. p.	↔ cell viability
	PPSPSY from in silico GID of garlic	150, 300, 600, 1200, and 2400 µM for 24 h	N. p.	↔ cell viability
	IASW from in silico GID of garlic	150, 300, 600, 1200, and 2400 µM for 24 h	N. p.	↔ cell viability
	WPQY from in silico GID of garlic	150, 300, 600, 1200, and 2400 µM for 24 h	N. p.	↔ cell viability
[31]	LGQQQPFPPQQPY from gliadin	100 µg/mL for 30 min, 3 and 6 h	N. p.	↔ cell viability
[39]	LGQQQPFPPQQPY from gliadin	100 µg for 24 h	N. p.	↓ cell viability; ↑ cell mortality
	LGQQQPFPPQQPY from gliadin and YDWPGGRN from wheat germ	100 µg for 24 h	N. p.	↓ cell viability; ↑ cell mortality
	LGQQQPFPPQQPY from gliadin and TGP from wheat germ	100 µg for 24 h	N. p.	↔ cell viability and mortality
	LGQQQPFPPQQPY from gliadin and QPYQQPQ from wheat germ	100 µg for 24 h	N. p.	↓ cell viability; ↑ cell mortality
[29]	CTLEW from walnut residual PH	0.5, 1, 2, 3, 4 mg/mL for 48 h	N. p.	↓ cell viability (IC_50_ = 0.65 mg/mL)
[20]	γ-EV from beans	1 mM for 2, 4, and 6 h	N. p.	↔ cell viability
		2.5, 3, 4, 5, and 10 mM for 2 h	N. p.	↓ cell viability at 10 mM
[21]	YPQPQ from malt hordein	10, 25, 50, 100, 200, and 400 μM for 2 and 4 h	N. p.	↓ cell viability
[32]	KLKKNL from *Salvia hispanica* seeds	0.75 (1009.48) and 1 mg/mL (1345.95 µM) for 24 h	N. p.	↑ Sub-G0 and G2/M phase; ↓ G1 phase; ↔ S phase
	MLKSKR from *Salvia hispanica* seeds	0.75 (948.27) and 1 mg/mL (1312.37 µM) for 24 h	N. p.	↑ Sub-G0 and G2/M phase; ↓ G1 phase; ↔ S phase
	KKYRVF from *Salvia hispanica* seeds	0.75 (892.83) and 1 mg/mL (1190.44 µM) for 24 h	N. p.	↑ Sub-G0; ↓ G1; ↓ S at 1 mg/mL; ↑G2/M at 0.75 mg/mL

Effects are referred to the respective unsupplemented control cells either in basal or stressed conditions. In the presence of more than one concentration/treatment time, only statistically significant changes are reported. When samples/concentrations/times are not reported, the effects are referred to all experimental conditions. ↑: increase; ↓: decrease; ↔: no effects; GID: (in vitro) gastrointestinal digested; N. p.: not present; PH: protein hydrolysate; RP-HPLC: reversed-phase high-performance liquid chromatography; SPE: solid phase extraction; TbOOH: tert butyl hydroperoxide.

**Table 8:** Concentration ranges used in the included Caco-2 studies versus an exploratory luminal benchmark, by functional domain.

Domain	Concentration Range Used	Exploratory Luminal Benchmark	Maximum Mass-Based Ratio	In Vivo Confirmation
Antioxidant	0.01–2.5 mg/mL for mixtures/fractions; 0.5–800 µM for defined peptides	~0.1–1 mg/mL for an aggregate candidate bioactive-peptide pool	~25× versus 0.1 mg/mL	None among the included studies
Anti-inflammatory	0.05–5 mg/mL; P31-43 tested at 100 µg/mL	~0.1–1 mg/mL for an aggregate candidate bioactive-peptide pool	~50× versus 0.1 mg/mL	None among the included studies
Antihypertensive	0.1–5 mg/mL for hydrolysates; 0.1–316 µM for defined peptides	Mass and molar ranges are not directly interchangeable	Not calculated for molar-only studies	Confirmed for the LTFPG metabolite in spontaneously hypertensive rats (external study)
Antidiabetic	0.5–5 mg/mL for hydrolysates/fractions; 1 µM–10 mM for defined peptides	~0.1–1 mg/mL for mass-based mixtures; sequence-specific exposure unknown	~50× versus 0.1 mg/mL for mass-based studies	None among the included studies
Cell viability/apoptosis	0.01–50 mg/mL for mixtures; 0.5 µM–10 mM for defined peptides	~0.1–1 mg/mL for an aggregate candidate bioactive-peptide pool	~500× versus 0.1 mg/mL	None among the included studies

## Data Availability

No new data were created or analyzed in this study. Data sharing is not applicable to this article.

## Supplementary Materials

The following supporting information can be downloaded at https://www.mdpi.com/article/10.3390/ijms27177905/s1.

## Abbreviations

The following abbreviations are used in this manuscript:

ACE	Angiotensin-converting enzyme
DPP-IV	Dipeptidyl peptidase-4
NF-κB	Nuclear factor-κB
NRF2	Nuclear factor erythroid 2-related factor 2
