# Supplementary material for: Biological Activity of Plant-Derived Peptides, Hydrolysates and Digests in the Caco-2 Intestinal Epithelial Model: A Systematic Critical Review
Source: Int J Mol Sci. 2026 Sep 4;27(17):7905. doi: 10.3390/ijms27177905 (PMC13566756; doi:10.3390/ijms27177905)
Supplement: Supplementary file 1 [file ijms-27-07905-s001.zip › ijms-4500348-supplementary.pdf]

Table S1. PRISMA 2020 checklist

Item numbering follows Page et al., BMJ 2021;372:n71. "Location" refers to the section of the revised manuscript; page numbers to be completed on the typeset proof.

#	Checklist item	Location in the revised manuscript
1	Title identifies the report as a systematic review	Title
2	Abstract: structured summary	Abstract
3	Rationale: description of the rationale in the context of existing knowledge	Introduction, paragraphs 1–4
4	Objectives: explicit statement of objectives or questions	Introduction, knowledge-gap paragraph and aim paragraph
5	Eligibility criteria: inclusion and exclusion criteria and grouping for synthesis	Section 2.1; Protocol S1
6	Information sources: all databases, registers and other sources; date of last search	Section 2.1 (PubMed, Scopus; 8 October 2025 and 4 May 2026); limitations 2–3 in Section 3.13
7	Search strategy: full search strategies for all sources	Section 2.1; Protocol S1, part 3; Table S5
8	Selection process: number of reviewers, independence, automation tools	Section 2.1 (two independent reviewers; no automation tools used)
9	Data collection process: number of reviewers, independence, automation tools	Section 2.1; Table S2
10a	Data items: all outcomes for which data were sought	Section 2.1, outcomes paragraph
10b	Data items: all other variables for which data were sought	Section 2.1; Table S2
11	Study risk of bias assessment: tool, number of reviewers, independence	Section 2.2 (QUIN, two independent reviewers); Table S4
12	Effect measures: effect measure used in synthesis or presentation	Section 2.3 (direction of effect; no pooled effect measure)
13a	Synthesis methods: process of deciding eligibility for each synthesis	Section 2.3, classification framework
13b	Synthesis methods: preparation of data for presentation or synthesis	Section 2.3 (no conversion or imputation performed)
13c	Synthesis methods: tabulation or visual display of results	Tables 1–8; Figures 2–5

#	Checklist item	Location in the revised manuscript
13d	Synthesis methods: methods used to synthesise results	Section 2.3, vote counting by direction of effect (Cochrane Handbook ch. 12)
13e	Synthesis methods: methods to explore heterogeneity	Section 2.3 (qualitative only; statistical exploration not possible)
13f	Synthesis methods: sensitivity analyses	Section 2.3 (no synthesis-level sensitivity analysis); a sensitivity analysis of the risk-of-bias scoring convention is reported in Section 2.2
14	Reporting bias assessment	Section 2.4 (not formally assessed; reasons given); limitation 3 in Section 3.13
15	Certainty assessment	Section 2.4 (GRADE not applied; reasons given); limitation 6 in Section 3.13
16a	Study selection: numbers screened, assessed and included, with flow diagram	Section 2.1; Figure 1
16b	Studies excluded at full text, with reasons	Section 2.1; Figure 1; Table S3
17	Study characteristics	Tables 2–8
18	Risk of bias in studies	Section 3.1; Figure 2; Table S4; Figure S1
19	Results of individual studies	Tables 2–7
20a–d	Results of syntheses	Sections 3.2–3.11; Tables 1 and 8; Figures 4–5
21	Reporting biases	Section 2.4; limitation 3 in Section 3.13
22	Certainty of evidence	Section 2.4; Sections 3.8 and 3.9 as substitute framework
23a–d	Discussion: interpretation, limitations of evidence and of review processes, implications	Sections 3.10–3.14; Section 4
24a	Registration and protocol: registration information	Section 2.1 and limitation 1 in Section 3.13 (not registered)
24b	Protocol access	Protocol S1 (prepared post hoc from the pre-screening agreement)
24c	Amendments to information provided at registration	Not applicable (no registration)
25	Support: financial and non-financial	Funding statement
26	Competing interests	Conflicts of Interest statement
27	Availability of data, code and other materials	Data Availability Statement; Supplementary Materials

Protocol S1. Eligibility criteria and screening rules

This protocol records the eligibility criteria and screening rules agreed between the two reviewers (S.P. and M.D.N.) before screening began. It was not prospectively registered; the limitation is stated in Section 3.13 of the manuscript. The text below is the operational form of the criteria as applied, including the definitions added during revision in response to reviewer comment.

1. Review question

What biological effects are produced by plant-derived peptides, protein hydrolysates and simulated gastrointestinal digests in Caco-2 cells; is the molecular species responsible for each reported effect identified; and are the tested concentrations plausibly attainable at the intestinal epithelium?

2. Eligibility criteria

Inclusion.

(i) Publication in English.

(ii) Report of a biological effect measured in Caco-2 cells of material derived from plant foods, ingredients, by-products or proteins, in any of four forms: (a) purified or synthetic single peptides (PP); (b) ultrafiltration or chromatographic fractions (FR); (c) whole protein hydrolysates or simulated gastrointestinal digests (HD); (d) intact parent proteins (IP), eligible only when tested as comparators for one of the preceding forms.

Exclusion.

(i) Plant-based food incorporated into a potentially animal-based food matrix.

(ii) Reviews, editorials, commentaries and conference abstracts.

(iii) Studies whose sole objective was quantification of transepithelial peptide transport, permeability or apparent permeability coefficients, with no biological-activity endpoint. Studies reporting a biological-activity endpoint alongside transport measurements were retained, and only the bioactivity data were extracted.

(iv) Studies in which the reported outcomes were generated exclusively in a co-culture system and could not therefore be attributed to Caco-2 cells alone. Where a study reported a Caco-2 monoculture arm alongside a co-culture arm, only the monoculture data were extracted.

(v) Inadequate statistical reporting, defined as the occurrence of any one of the following: (a) no inferential test reported for the comparison between control and treated conditions; (b) no measure of dispersion reported for the compared groups; (c) the number of independent biological replicates not stated, or fewer than three; (d) a claim of statistical significance without identification of the test applied.

(vi) Insufficient methodological detail to reconstruct the experimental conditions, specifically the absence of any one of: identity of the tested material, concentration and unit, exposure duration, assay used.

(vii) Genetically modified Caco-2 cells, or chemically modified peptides.

3. Search strategy

Databases: PubMed and Scopus. String: “food bioactive peptides” AND “cultured cells”. First search executed 8 October 2025 with no lower date limit; update search executed 4 May 2026 restricted to records from 9 October 2025 onwards. No language filter was applied at retrieval; English-language restriction was applied at screening.

No automation tool, machine-learning classifier or text-mining software was used at any stage. Backward citation chasing was performed on every full text retrieved for detailed evaluation.

The quotation marks were entered as phrase-search operators. They did not, however, produce an equivalent operation in the two interfaces: PubMed resolves a quoted string as an exact phrase only when that string is present in its phrase index and otherwise dissolves it into its component terms for Automatic Term Mapping, whereas double quotation marks in Scopus denote a loose phrase search rather than the exact-string match denoted by braces. The verbatim strings as submitted, the fields interrogated, the filters and date limits applied and the records retrieved are given in Table S5.

Rationale for the two-concept string, and for the choice of databases, is given in Section 2.1 of the manuscript; the residual risk of missed records is stated as limitations 2 and 3 in Section 3.13.

4. Screening procedure

Both reviewers screened titles independently, then abstracts, then full texts. Disagreements at each stage were resolved by discussion; no third adjudicator was required. Original investigators were not contacted for additional information. No assumptions were made about missing or unclear information: studies with insufficient methodological detail were excluded under criterion (vi). Every full text not retained was recorded with the specific criterion applied; the list is given as Table S3.

5. Classification frameworks applied to included studies

Material class (a study may carry more than one label): PP, purified or synthetic single peptide; FR, ultrafiltration or chromatographic fraction; HD, whole protein hydrolysate or simulated gastrointestinal digest; IP, intact parent protein.

Characterisation of the bioactive species (mutually exclusive; where more than one description applied, the study was assigned to the category reflecting the most advanced characterisation achieved):

Category	Definition
A	A breakdown metabolite was identified and independently tested.
B	An uncharacterised fraction or hydrolysate was tested; the active component was not isolated.
C	A single purified or synthetic peptide was tested directly; its stability in the assay system was not assessed.
D	A simulated gastrointestinal digestion was applied, but brush-border-generated products were not characterised.
E	The tested sequence is characterised in the independent literature as resistant to intestinal digestion.
F	An intact parent protein was the primary material tested.
G	An intact protein and its gastrointestinal digest were directly compared.

6. Synthesis rule

Vote counting by direction of effect (increase, decrease, no significant change relative to each study's own control). Every count reported in the manuscript text is computed at the level of the study and never at the level of the experimental condition: each study contributes exactly one vote per outcome domain in which it reports a result, irrespective of the number of concentrations, exposure times or fractions tested. No pooled effect measure, heterogeneity statistic, subgroup analysis, meta-regression or synthesis-level sensitivity analysis was computed, and no data conversion or imputation was performed.

Table S2. Data-extraction template

Fields extracted independently and in duplicate by both reviewers. The form was piloted on five randomly selected studies before use.

Field	Definition and permitted values
Reference ID	Bibliographic identifier assigned in the review
Plant source	Botanical species or common name of the source material
Food matrix	Seed, flour, by-product, beverage, isolate, etc.
Material class	PP / FR / HD / IP (multiple labels permitted)
Tested material, as described	Verbatim description used by the original authors
Peptide sequence	One-letter code where identified; “not identified” otherwise
Molecular weight or size cut-off	Da or kDa; ultrafiltration cut-off where applicable
Digestion protocol	INFOGEST, other named protocol, in silico, or none
Concentration tested	Value and unit exactly as reported; no inter-conversion
Exposure duration	Minutes or hours as reported
Exogenous stressor	Agent and concentration, or “not present”
Cell state	Differentiated / undifferentiated / early-confluent, where stated
Outcome domain	Antioxidant / anti-inflammatory / antihypertensive / antidiabetic / other / viability (multiple permitted)
Assay	Named assay or probe
Direction of effect	Increase / decrease / no significant change, versus the study's own control
Quantitative value reported	IC50, percentage change, etc., reproduced as given
Statistical test reported	Named test; measure of dispersion; number of independent replicates
Bioactive-species characterisation	Category A–G
Metabolic-fate analysis performed	Yes / no; if yes, method and product identified
Non-peptide control	Yes / no (e.g. phenol-depleted comparator)
Viability control at each concentration	Yes / no
Extractor and date	Initials of the reviewer; date of extraction
Discrepancy and resolution	Free text; resolved by discussion

Table S3. Full texts assessed for eligibility, with reasons for exclusion

Twenty records were retrieved in full text and not retained: 17 at the full-text stage of the first search (8 October 2025) and 3 at the full-text stage of the second search (4 May 2026). These counts reconcile with the PRISMA flow diagram in Figure 5. Each record is listed with the single criterion that determined its exclusion; where more than one criterion applied, the first encountered in the order below was recorded.

Code	Criterion applied (Protocol S1, part 2)
IN-i	Inclusion criterion (i) not met: not published in English
IN-ii	Inclusion criterion (ii) not met: no biological effect measured in Caco-2 cells, or material not of plant origin
EX-i	Plant-based food incorporated into a potentially animal-based food matrix
EX-ii	Review, editorial, commentary or conference abstract
EX-iii	Transepithelial transport, permeability or Papp as the sole endpoint, with no biological-activity endpoint
EX-iv	Outcomes generated exclusively in a co-culture system
EX-v	Inadequate statistical reporting (see Protocol S1 for the operational definition)
EX-vi	Insufficient methodological detail to reconstruct the experimental conditions
EX-vii	Genetically modified Caco-2 cells or chemically modified peptides

Table S4. Per-study QUIN scoring worksheet

Scoring: 0 = not specified; 1 = inadequately specified; 2 = adequately specified; N.a. = not applicable. Final score = (total score × 100) / (2 × number of applicable criteria). Sampling technique is not applicable throughout, so 11 of the 12 criteria are applicable and the denominator is 22. Risk of bias: low > 70%, medium 50–70%, high < 50%. As stated in Sections 3.1 and 2.2 of the manuscript, the majority of QUIN items assess completeness of reporting rather than demonstrated bias; scores are to be read as risk of bias arising from incomplete reporting.

Domains D1, D2 and D4–D7 took the same value for all 34 studies and are pre-entered. Domain D3 (sampling technique) is N.a. for all studies and is therefore excluded from the table and from the denominator. Domains V1–V5 vary between studies and must be transferred from the scoring file used to generate Figure S1; the total and the final score follow arithmetically once they are entered. Randomization, blinding, operator details and outcome-assessor details are scored 0 rather than N.a.; the rationale and a sensitivity analysis of the effect of this convention on the classification are given in Section 2.2 of the manuscript.

Code	QUIN domain	Status in this review
D1	Details of comparison group	2 — adequately specified in all 34 studies
D2	Method of measurement of outcome	2 — adequately specified in all 34 studies
D3	Detailed explanation of sampling technique	N.a. — not applicable in all 34 studies; excluded from the denominator
D4	Operator details	0 — not specified in any of the 34 studies
D5	Randomization	0 — not specified in any of the 34 studies
D6	Outcome assessor details	0 — not specified in any of the 34 studies
D7	Blinding	0 — not specified in any of the 34 studies
V1	Clearly stated aims / objectives	Varies between studies
V2	Detailed explanation of sample size calculation	Varies between studies
V3	Detailed explanation of methodology	Varies between studies
V4	Statistical analysis	Varies between studies
V5	Presentation of results	Varies between studies

Table S5. Database-specific search syntax

Strings are given exactly as submitted to each interface. Both searches were run by the same two reviewers on the dates shown. No automation tool, machine-learning classifier or text-mining software was used at any stage.

Round	Database	Interface and fields	String as entered	Date run	Filters and date limits	Records retrieved
1	PubMed	Basic search box; no field tags applied	"food bioactive peptides" AND "cultured cells"	8 Oct 2025	None; no lower date limit; no language filter	1552
1	Scopus	Advanced search; TITLE-ABS-KEY field	TITLE-ABS-KEY("food bioactive peptides" AND "cultured cells")	8 Oct 2025	None; no lower date limit; no language filter	45
2	PubMed	Basic search box; no field tags applied	"food bioactive peptides" AND "cultured cells"	4 May 2026	Publication date from 9 Oct 2025 onwards	143
2	Scopus	Advanced search; TITLE-ABS-KEY field	TITLE-ABS-KEY("food bioactive peptides" AND "cultured cells")	4 May 2026	Publication date from 9 Oct 2025 onwards	5

Records after removal of duplicates: 1561 in round 1 and 147 in round 2. Backward citation chasing of every full text retrieved for detailed evaluation added 4 records in round 1 and 1 in round 2. Total included: 34.

Figure S1. Per-study QUIN traffic-light plot

Per-study appraisal of the 34 included publications across the eleven applicable QUIN domains, displayed in the traffic-light format. Green, adequately specified (2); amber, inadequately specified (1); red, not specified (0). Sampling technique is not applicable throughout and is not displayed. The figure presents the QUIN domains actually scored and does not imply that the Cochrane Risk of Bias 2 tool was applied.

Figure S1. Per-study QUIN appraisal of the 34 included studies

● 2 - adequately specified ● 1 - inadequately specified ● 0 - not specified ● not applicable

Study	1. Clearly stated aims/objectives	2. Sample-size calculation	3. Sampling technique	4. Details of comparison group	5. Detailed explanation of methodology	6. Operator details	7. Randomization	8. Method of outcome measurement	9. Outcome assessor details	10. Blinding	11. Statistical analysis	12. Presentation of results
Amigo-Benavent [12]	+	+	n/a	+	±	−	−	+	−	−	±	±
Bollati [[13]	+	±	n/a	+	+	−	−	+	−	−	±	±
Capraro [[14]	+	±	n/a	+	±	−	−	+	−	−	+	±
Cheng [15]	+	±	n/a	+	±	−	−	+	−	−	±	+
Cruz-Chamorro [16]	±	+	n/a	+	+	−	−	+	−	−	±	±
Dellafiora [17]	±	+	n/a	+	±	−	−	+	−	−	+	±
Dugardin [18]	±	+	n/a	+	±	−	−	+	−	−	±	+
García-Nebot [19]	±	±	n/a	+	+	−	−	+	−	−	+	±
Guha [20]	+	+	n/a	+	+	−	−	+	−	−	±	±
He [21]	+	+	n/a	+	±	−	−	+	−	−	+	±
Hu [22]	+	+	n/a	+	±	−	−	+	−	−	±	+
Lammi [23]	+	±	n/a	+	+	−	−	+	−	−	+	±
Lammi [24]	+	±	n/a	+	+	−	−	+	−	−	±	+
Lammi [25]	+	±	n/a	+	±	−	−	+	−	−	+	+
Lammi [26]	±	+	n/a	+	+	−	−	+	−	−	+	±
Liang [27]	±	+	n/a	+	+	−	−	+	−	−	±	+
Lopes [28]	+	+	n/a	+	+	−	−	+	−	−	+	±
Ma [29]	+	+	n/a	+	+	−	−	+	−	−	±	+
Montserrat-de la Paz [30]	+	+	n/a	+	±	−	−	+	−	−	+	+
Nanayakkara [31]	+	±	n/a	+	+	−	−	+	−	−	+	+
Quintal Bojórquez [32]	±	+	n/a	+	+	−	−	+	−	−	+	+
Rasera [33]	+	+	n/a	+	+	−	−	+	−	−	+	±
Roytrakul [34]	+	+	n/a	+	±	−	−	+	−	−	+	+
Tonolo [35]	+	+	n/a	+	+	−	−	+	−	−	+	+
Tonolo [36]	+	+	n/a	+	+	−	−	+	−	−	+	+
Urbizo-Reyes [37]	+	+	n/a	+	+	−	−	+	−	−	+	+
Vilcacundo [38]	+	+	n/a	+	+	−	−	+	−	−	+	+
Wang C. [39]	+	+	n/a	+	+	−	−	+	−	−	+	+
Wang L. [40]	+	+	n/a	+	+	−	−	+	−	−	+	+
Xie [41]	+	+	n/a	+	+	−	−	+	−	−	+	+
You [42]	+	+	n/a	+	±	−	−	+	−	−	±	±
Zhang Q. [43]	+	±	n/a	+	+	−	−	+	−	−	±	±
Zhang Q. [44]	+	±	n/a	+	±	−	−	+	−	−	+	±
Zhang T. [45]	+	±	n/a	+	±	−	−	+	−	−	±	+

Score = (total × 100) / (2 × number of applicable criteria); 11 criteria applicable, denominator 22. Risk of bias: low > 70%, medium 50-70%, high < 50%.
